# Statistical Investigation and Optimization of Starch Cinnamylation: A Design of Experiment Approach

**DOI:** 10.1021/acsomega.5c13678

**Published:** 2026-04-10

**Authors:** Luca Leuzzi, Laura Cipolla

**Affiliations:** † Department of Biotechnology and Biosciences, 9305 University of Milano-Bicocca, Piazza della Scienza 2 20126, Milan,Italy

## Abstract

The investigation and optimization of the cinnamylated starch degree of substitution (DS) in basic media were carried out using a statistical approach based on the design of experiment (DoE) approach. An initial screening phase, conducted through a full factorial design, highlighted the following as the most critical parameters affecting the DS: (i) the base amount, (ii) the nature of the alkyl halide, (iii) the addition mode to the reaction mixture, and (iv) the equivalents used. The efficacy of different bases promoting the alkylation reaction was also studied, comparing sodium hydroxide, sodium alkoxides, and the dimsyl ion. Overall, the screening phase identified cinnamyl chloride and a sodium hydroxide DMSO dispersion as the optimal starting points for the optimization phase. The resulting model proved to be a robust tool for identifying optimal reaction conditions to achieve a high DS. The DoE statistical studies provided valuable insights into the mechanistic aspects and the complexity of the reaction system, extending beyond starch functionalization. These findings represent a significant advancement in the synthesis of starch-based materials, particularly for applications in the development of UV radiation-sensitive compounds, since they hold considerable potential across various industrial sectors.

## Graphical abstract

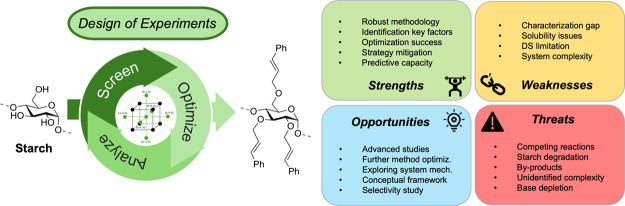

## 1 Introduction

In recent decades, the adoption of bioplastics, particularly biodegradable plastics, has significantly increased across various sectors. This trend is largely attributed to the urgent need for environmentally sustainable alternatives to traditional nonbiodegradable petrochemical products, given the alarming rise in global plastic consumption and associated waste.^1^ Additionally, the production processes for petroleum-based plastics often involve the utilization of hazardous chemicals, posing risks not only to the health and safety of human workers but also to the broader environmental ecosystem. Furthermore, the dispersion of microplastic debris resulting from these production methods exacerbates the ecological impact of conventional plastics.^2^

It is essential to clarify that the term “bioplastic” serves as a broad classification encompassing both biobased and biodegradable plastics. In the context of biobased plastics, this designation refers to materials that are derived from biological or renewable sources; however, this does not inherently imply that they possess biodegradable properties. For a material to be classified as biodegradable, it must exhibit the ability to decompose into biomass, carbon dioxide, and water through interactions with biological agents, such as microorganisms.^3,4^ In this framework, starch emerges as a prominent biobased material for the synthesis of biodegradable polymers.^5^ Despite facing mechanical challenges, such as brittleness and hydrophilicity associated with pure starch films,^6^ this polysaccharide has been extensively investigated in the literature for its application in the production of packaging materials and various other uses.^7^ The advantages of starch as a feedstock can be attributed to its abundant availability, cost-effectiveness, and relatively environmentally safe processing methods.^8^

To address the limitations of native starch, modifications can be implemented through chemical^9^ or physical^10^ processes or blending it with biodegradable synthetic polymers enhancing its functional properties.^11^ However, it is important to note that many reagents utilized in their chemical modifications raise significant safety and health concerns, particularly in applications such as food packaging. Consequently, there is a strong preference for the use of naturally derived reagents to ensure the process and byproducts remain as safe as possible.^12^

In this context, the [2 + 2] reversible photo cycloadditions can be considered as a real promising starting point.^13^ Among the substrates that can actively participate in these kinds of reactions, cinnamic acid and derivatives^14,15^ are discussed, thanks to their natural properties and ability to act as a cross-linker after being grafted onto starch’s backbone. The photocross-linking process can be actively exploited to manipulate polymers’ properties under irradiation exposure.^16^ However, the existing literature reveals a notable deficiency in information regarding the utilization of cinnamyl-functionalized starch as a precursor for the production of biobased films through reversible photochemical cycloaddition processes. Additionally, the degree of substitution (DS) of a polymer with UV-reactive moieties plays a crucial role in determining the efficacy of the subsequent photocross-linking. In this framework, the present study investigates the synthetic methodology previously delineated by our research group^17^ for the preparation of starch cinnamyl ethers, employing a DoE (design of experiment) approach, a well-known analytical procedure in industry and research.^18–20^ The main goal is to identify the significant procedure variables that can subsequently be used in optimizing the functionalization process.

The etherification reaction was investigated through a comprehensive and systematic approach, initially from a theoretical perspective and subsequently through the experimental variation of multiple parameters. An in-depth analysis of the reaction and its mechanistic complexity led to the identification of potential key factors influencing the outcome. Based on these insights, a detailed statistical experimental study was performed (Scheme 1). A screening phase designed to isolate the most critical variables was conducted via a series of three sequential DoE steps (SP1-DoE, SP2-DoE, and SP3-DoE). Importantly, the models were utilized iteratively rather than simultaneously or through a singular comprehensive model. This approach allowed for progressively refined insights and a more effective interpretation of the experimental data. This segment of the study investigated: (i) the selection of the base and cinnamyl halide identities, (ii) the equivalents of the base and the alkylating agent with respect to starch, (iii) the substrate concentration, (iv) the temperature employed during the deprotonation step, and (v) the methodology of cinnamyl halide addition. The insights gathered from these investigations informed the optimization phase, during which the DS was studied as a function of the equivalents of both the base and the alkylating agent. Proton nuclear magnetic resonance (^1^H NMR) spectroscopy was used to determine the DS.

**Scheme 1:**
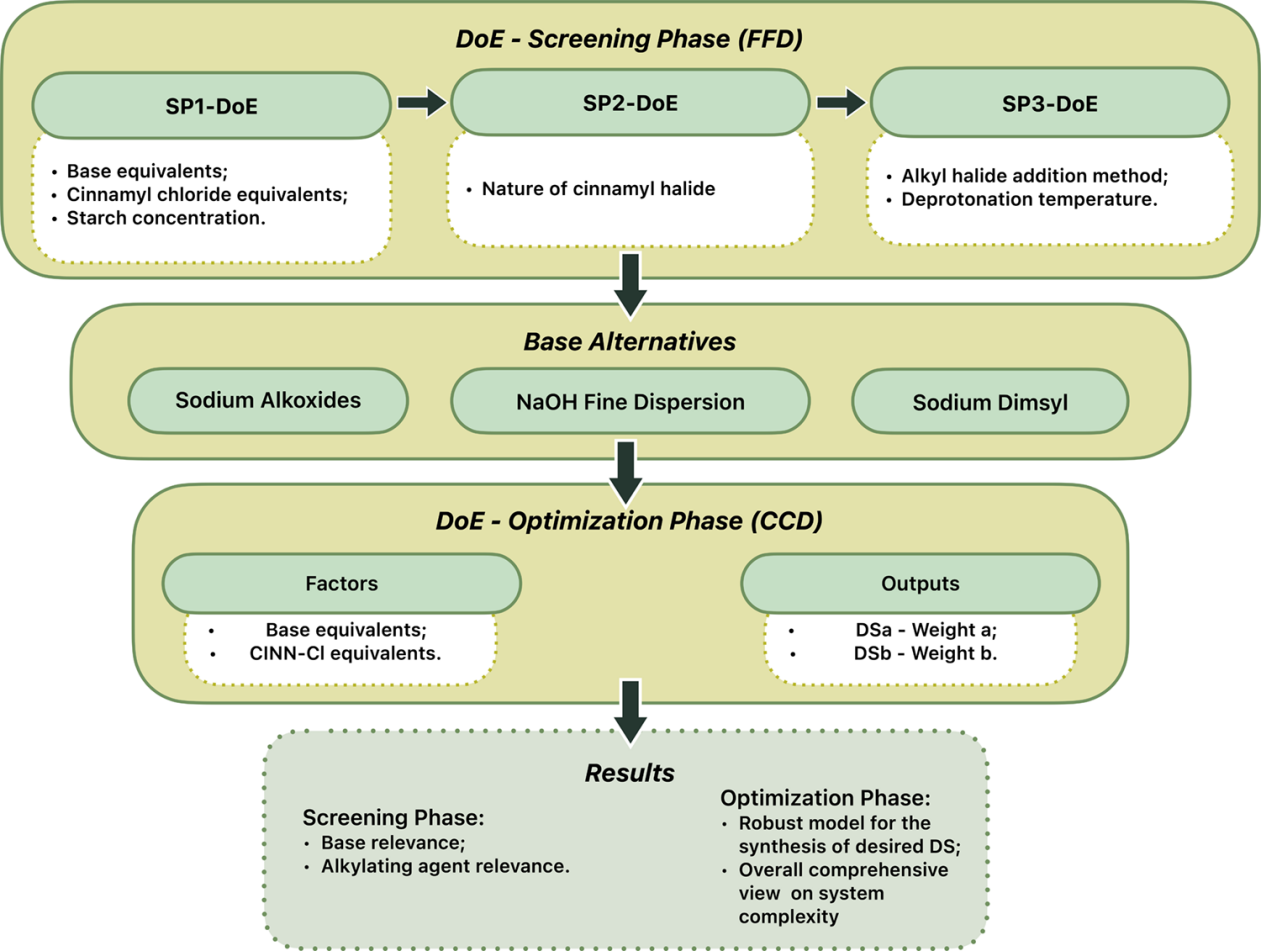
Methodological Flowchart Illustrating the Process Adopted and Described in the Manuscript with the Studied Factors and the Obtained Results

We hypothesize that the equivalents and the identity of the base, along with the alkylating agent, will emerge as the dominant parameters influencing the overall DS of starch cinnamylation. We predict that these factors will demonstrate complex, nonlinear (quadratic) and linear relationships that, when combined through statistical optimization, will enable achieving a DS significantly maximized compared to our previous studies.

To the best of our knowledge, this represents the first time that such a statistical and detailed approach has been employed to study starch etherification with cinnamyl halides.

## 2 Experimental Section

### 2.1 Materials and Methods

Native potato starch powder (CAS 9005-25-8, purity grade ≥ 99%, DB(%) = 3.631% (Figure S6), Mw = 2.37 × 10^6^ Da (Section S4, Figure S5)), kindly gifted by Novidon B.V. (Handelsweg 36-38, Nijmegen, The Netherlands), was dried in a static oven at 65–70°C for at least 48 h before use; the average weight loss was 16.625% ± 0.093. Predominantly trans cinnamyl bromide–(3-bromophenyl) benzene (CINN-Br, CAS 4392-24-9, purity 97%) was purchased from Acros Organics. Trans cinnamyl chloride–(3-chloropropenyl) benzene (CINN-Cl, CAS 2687-12-9, purity 95%), anhydrous NaOH (pellets, CAS 1310-73-2, purity grade ≥ 98%), sodium hydride (NaH, CAS 7646-69-7, 60% dispersion in mineral oil), benzoyl chloride (PhCOCl, CAS 98-88-4, purity 99%), 1-allyl-3-methylimidazolium chloride (AMIM-Cl, CAS 65039-10-3, purity ≥ 97%), and anhydrous pyridine (Py, CAS 110-86-1, purity 99.8%) were purchased from Sigma-Aldrich. Acetone (CAS 67-64-1, purity 96.6%), anhydrous dimethyl sulfoxide (DMSO, CAS 67-68-5, purity ≥ 99.7%, extra-dry), chloroform (CHCl_3_, CAS 67-66-3, purity ≥ 99.8%) of Honeywell Riedel-de Haën, absolute ethanol (EtOH, CAS 64-17-5, purity ≥ 99.8%), and sodium (CAS 7440-23-5, purity 99.8% oiled sticks) were purchased from Thermo Fisher Scientific. Methanol (MeOH, CAS 67-56-1, HPLC PLUS grade) was purchased from Carlo Erba. 2-Propanol (CAS 67-63-0, purity ≥ 99.8%) was purchased from Honeywell Riedel-de Haën. Before use, the NaOH pellets were crushed and pulverized in a mortar. All of the other reagents and solvents were used as provided and without further purification. Ultrapure water was obtained using the Milli-Q system with a residual conductivity of 13 μS·cm^–1^.

DMSO-*d*_6_ (CAS 2206–27–1, 99.5 atom % D) and trifluoroacetic acid-*d* (TFA-*d*, CAS 599–00–8, 99.5 atom% D) were purchased from Acros Organics, while chloroform-*d* (CDCl_3_, CAS 865–49–6, 99.8 atom % D) was purchased from Sigma-Aldrich. Thin-layer chromatography (TLC) was performed on silica gel 60 F_254_ precoated glass plates (Merck), using a 10:0.5 CHCl_3_:EtOH mixture as the eluent and visualized using a UV lamp at a wavelength of 254 nm. Flash column chromatography was performed using silica gel (60 Å, particle size 40–64 μm) as the stationary phase, following the procedure by Still and co-workers.^21^

### 2.2 General Procedures for the Synthesis of Starch Cinnamyl Ethers in the Screening Phase (SP1–3-DoE)

Starch/base/cinnamyl halide ratios were calculated by considering the starch in terms of anhydroglucopyranose unit (starch-AGU, Mw = 162.14 g·mol^–1^). Typically, 500 mg (3.08 mmol of starch-AGU) or 200 mg (1.23 mmol) of dried potato starch were, respectively, suspended in 8.33 or 10 mL of dry DMSO (respectively, [starch-AGU] = 60 g·L^–1^ or 20 g·L^–1^) in a round-bottom flask (25 mL) and heated at 80–90 °C, thanks to an oil bath under mild magnetic stirring, until complete dissolution (3 h). The solution was then cooled down to room temperature, and the base (1.0 to 3.0 equiv) was added directly into the main batch, which was left to homogenize for 1 h under magnetic stirring. Afterward, the cinnamyl halide (1.0 to 3.0 equiv) was carefully added, and the mixture was stirred magnetically for 24 h at room temperature, keeping the flask in the dark. Based on the reagent quantities, the final batch ranged from slightly yellow to dark red color. After 24 h, the mixture was then slowly poured into freezer-cold acetone (15 mL) in a plastic centrifuge tube, and gently stirred. After 10 min, the suspension was centrifuged (5000 rpm, 15 °C, 10 min), the solvents carefully removed, and the solids washed with a mixture of DI-water:acetone in a 3:30 ratio in order to get rid of the residual organics (reagents and byproducts) and inorganics (bases and salts). The suspension was centrifuged again (5000 rpm, 15 °C, 10 min), the solvents removed, and the precipitate washed with fresh acetone (20 mL or 2 × 20 mL if needed). The precipitate was then finally recovered by centrifugation (5000 rpm, 15 °C, 10 min), solvent removal, and air drying in the fume hood. TLC of wash waste was used to monitor the efficiency of the process (mobile phase: 10:0.5 - CHCl_3_:EtOH; *R*_*f*(CINN‑Cl)_: 0.98, *R*_*f*(CINN‑OH)_: 0.50).

### 2.3 General Procedures for the Synthesis of Starch Cinnamyl Ethers in the Optimization Phase

Starch/base/cinnamyl halide ratios were calculated by considering the starch in terms of anhydroglucopyranose unit (starch-AGU, Mw = 162.14 g·mol^–1^). Dried potato starch (500 mg, 3.08 mmol of starch-AGU) was suspended in dry DMSO ([starch-AGU] = 60 g·L^–1^) in a round-bottom flask (25 mL) and heated at 80–90 °C, thanks to an oil bath under mild magnetic stirring, until complete dissolution (2 h). The solution was then cooled down to room temperature, and the dispersed base (*vide infra*) in dry DMSO (0.586 to 3.414 equiv) was added directly into the main batch, which was left to homogenize for 1 h under magnetic stirring (in case of strong initial gelatinization hampering magnetic stirring, manual aid was needed). Afterward, cinnamyl chloride (0.586 to 3.414 equiv) was carefully added portion-wise to the main batch, and the mixture was stirred magnetically for 24 h at room temperature, keeping the flask in the dark. Based on the reagent quantities, the final batch ranged from slightly yellow to dark red color. After 24 h, the mixture was then slowly poured into freezer-cold acetone (15 mL) in a plastic centrifuge tube, and gently stirred. After 10 min, the suspension was centrifuged (5000 rpm, 15 °C, 10 min), the solvents carefully removed, and the solids were washed with a mixture of DI-water:acetone in a 3:30 ratio in order to get rid of the residual organics (reagents and byproducts) and inorganics (bases and salts). The suspension was centrifuged again (5000 rpm, 15 °C, 10 min), the solvents removed, and the precipitate washed with fresh acetone (20 mL or 2 × 20 mL if needed). The precipitate was then finally recovered by centrifugation (5000 rpm, 15 °C, 10 min), solvent removal, and air drying in the fume hood. Into the first wash’s waste liquor was added 50 mL of acetone; a second precipitate was recovered and washed as described above. TLC of wash waste was used to monitor the efficiency of the process (mobile phase: 10:0.5 - CHCl_3_:EtOH; *R*_*f*(CINN‑Cl)_: 0.98, *R*_*f*(CINN‑OH)_: 0.50).

Additional procedures, characterizations, and statistical analyses are reported in the Supporting Information.

## 3 Results and Discussion

The main scope of this work is the in-depth study of the effect of different functionalization reagents and conditions toward maximizing the DS, based on a cinnamyl starch functionalization reaction proposed by our research group^17^ (Scheme 2). The proposed procedure was centered on the use of NaOH (sodium hydroxide) as base to deprotonate the starch’s hydroxy groups and CINN-Cl (cinnamyl chloride) as alkylating agent in anhydrous DMSO (dimethylsulfoxide) as reaction media.

**Scheme 2:**
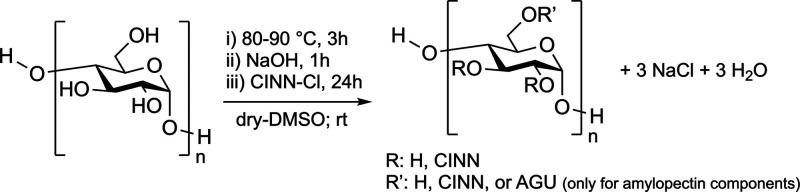
Starch Functionalization Exemplified on a Single Starch-AGU Unit

Herein, the starch etherification reaction with cinnamyl halides is explored through a DoE approach in order to evaluate the process variables and gain some hints about the mechanisms and the reactivity behind such a complex system.

### 3.1 Characterization of Potato Starch

Considering that the functionalization of starch is profoundly influenced by its polysaccharide composition and structural features, an initial characterization of potato starch was conducted. Despite being represented by its glucosidic repeating unit (starch-AGU, anhydroglucopyranose unit) in Scheme 2, starch is a complex polysaccharide with a certain DB (degree of branching). This characteristic distinguishes its two primary components, amylose and amylopectin, which are connected by α-1,4- and α-1,6-glycosidic bonds. Therefore, potato starch DB% was determined by ^1^H NMR (Figure S6) as described in the Supporting Information (Section S1.2.2, alongside Mw determination - Section S4) based on the anhydroglucopyranose unit, and resulted in 3.631%, which can be converted into a maximum achievable DS of 2.964.

### 3.2 Analysis of Reaction and Mechanism Complexity

To optimally configure the DoE and, more importantly, identify the factors that need to be studied, it is essential to analyze first the reaction mechanism from a theoretical perspective. The presence of NaOH is crucial for the deprotonation of starch, resulting in the formation of the corresponding alkoxide. The alkoxide can act as a nucleophile (Nu) engaging an S_N_2 reaction on the alkylating agent (CINN-X), given that the nucleophiles involved are either primary or secondary, and the halide comprises a primary carbon with a good leaving group (Williamson’s etherification). For each reacted hydroxy group, a mole of water and a sodium halide is produced. This theoretical framework assumes ideal conditions, which, unfortunately, is often not representative of reality, particularly when considering potential side reactions, polyfunctional molecules raising regiochemical issues, topochemical effects, and reagent solubility issues in the reaction media.

First, it is important to recognize that the base is actively consumed during the reaction, leading to the formation of sodium salts and water. Consequently, the quantity of base present in the reaction mixture can already be considered as a critical parameter, affecting the theoretical DS. It is worth noting that the hydroxide ion may also participate as a nucleophile in the substitution reaction, affording CINN-OH (cinnamyl alcohol) as a byproduct. This phenomenon not only results in the excessive consumption of both the base and the substrate but also yields a base-reactive byproduct, CINN-OH itself, which can undergo deprotonation and function as an additional nucleophile, potentially promoting the formation of dicinnamyl ether (CINN-O-CINN). Based on these observations, it can be speculated that in the worst-case scenario, two moles of NaOH and CINN-X would be irretrievably consumed and would not react with starch. Another important key point is the actual difference of p*K*_a_ values among the starch hydroxy groups and the base; generally speaking, for an effective deprotonation, it is recommended to have at least a Δp*K*_a_ > 4/5; in water, starch p*K*_a_ is reported to be around 12.5,^22^ while for NaOH, it is 14; however, it is expected that in DMSO, the p*K*_a_ will increase because the sodium ion is coordinated by the solvent. Thus, in the discussed case, the difference should almost reach the desired value; nonetheless, the use of a stronger base could enhance the deprotonation process.

The reactivity of the system may also be influenced by steric effects: the bulkiness hindrance may arise during the progressive functionalization of the polysaccharide and/or in the case of hindered position at carbon 6 due to branching chains (amylopectin), which further constrains the spatial environment surrounding the polysaccharide backbone. Despite the hydroxy groups being naturally arranged in the equatorial positions, the functionalization process may significantly limit the available space for additional reactions. Moreover, the presence of the cinnamyl moiety, characterized by an aromatic ring, introduces a degree of rigidity due to conjugative effects, thereby further restricting molecular accessibility (Figure S2). Consequently, for positions 2 and 3, it might be speculated that it will be necessary to further push the reaction conditions to favor vicinal etherification, considering the potential steric hindrance that might impede this modification. Furthermore, it is important to acknowledge that both dry starch and sodium hydroxide exhibit hygroscopic properties, which may lead to the presence of additional water molecules due to moisture absorption. These water molecules could potentially interfere with the mechanism acting as nucleophiles or initiating undesirable side reactions. Finally, the physical properties of starch must be carefully considered, as the gelatinization^23,24^ process may hinder the reaction due to variations in viscosity within the reaction media.

The reported thorough analysis of the reaction system enabled the identification of the factors considered significant in the DoE.

### 3.3 Screening Phase (SP)–Full Factorial Design

#### 3.3.1 SP1-DoE–(NaOH/CINN-Cl/Concentration)

To perform the first screening phase (SP1-DoE) on the role of the reaction variables, a full factorial design (FFD) was performed with a complete focus on the mathematical model construction, studying the main effects and interactions. A two-level model was considered in order not to over-increase the number of experiments to be performed (Figure 1). Usually, a reasonable number of factors for an FFD is three or four, but in this last case, the number of experiments may be excessively high, and a fractional factorial design (FrFD) would be necessary. In the screening phase, in all the FFD designs (SP1, SP2, and SP3-DoE), replicates were deliberately excluded, as they would not increase the degrees of freedom (DoF), and consequently return no information about the associated error, as indicated by the following equation:

DoF=N−P−R

where “N” is defined as the number of experiments performed, “P” represents the number of terms in the model equation, and “R” indicates the number of replicates. The software suggested a total of 8 and 4 experiments when considering three and two factors, respectively, based on the formula 2^*k*^, where the term k represents the number of factors.

**Figure 1:**
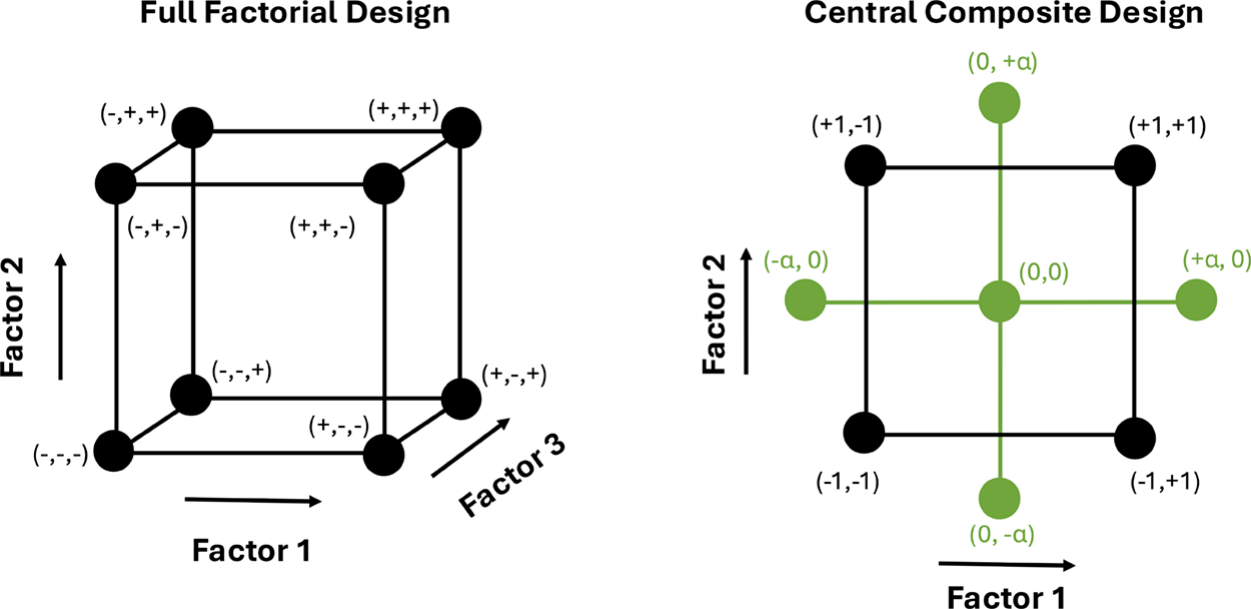
Schematic representation of a 3-factor 2-level FFD, and a 2-factor 2-level CCD constituted by a FFD (in black) merged with a SD (star design) (in green).

In our specific case, for the 3-factor FFD:

DoF=8−8−0=0

and for the 2-factor FFD:

DoF=4−4−0=0

Based on the previous knowledge of the research group and the reaction system analysis reported in Section 3.2, amounts of base, cinnamyl chloride, and starch concentration in the reaction media were the factors of choice for the SP1-DoE. The levels were coded with “ ± 1” values (Table S2). In particular, referring to the reagents, “–1” represents 1.0 equivalents, and “+1” represents 3.0 equivalents. These values are based on the assumption that 1.0 equivalent (equiv) is the stoichiometric amount of reagents that are needed to functionalize a single hydroxy group of the starch-AGU unit. Meanwhile, for the concentration factor, the values oscillate between 60 g·L^–1^ and 20 g·L^–1^, respectively, for “+1” and “–1”. Therefore, the JMP Pro^25^ (JMP Pro, Version 18.0.2, 2024) software was set up for a two-level three-factor FFD (2^3^ FFD) with all continuous factors obtaining a specific matrix (Table S3). Taking into account the codification, and performing all the tests in a randomized order, after the experiments, all the collected data are reported in Table 1, where the output of the model is represented by the final DS.

**Table 1:** FFD Matrix with the Outputs^a^

entry	NaOH (equiv)	CINN-X (equiv)	conc (g·L^–1^)	DS^b^
1	3.0 [+1]	1.0 [−1]	60 [+1]	0.55
2	1.0 [−1]	3.0 [+1]	60 [+1]	0.02
3	3.0 [+1]	3.0 [+1]	60 [+1]	0.34
4	3.0 [+1]	3.0 [+1]	20 [−1]	0.20
5	1.0 [−1]	1.0 [−1]	20 [−1]	0.02
6	1.0 [−1]	1.0 [−1]	60 [+1]	0.01
7	1.0 [−1]	3.0 [+1]	20 [−1]	0.02
8	3.0 [+1]	1.0 [−1]	20 [−1]	0.32

a The corresponding coded values are reported in the square brackets.

b Determined by ^1^H NMR.

The model returned an equation containing a total of 8 coefficients (eq S4, Section S1.4.1), including the intercept and the interaction coefficients. Specifically, the estimated effects for each parameter evaluated are reported in Table 2.

**Table 2:** Estimated Effects of FFD

factors	coeff.	pseudo *p*-value^a^
intercept	0.186	
[NaOH]	0.167	0.0955
[CINN-Cl]	–0.040	0.5739
[Conc]	0.046	0.5231
[NaOH*CINN-Cl]	–0.042	0.5559
[NaOH*Conc]	0.049	0.4939
[Conc*CINN-Cl]	–0.011	0.8688
[NaOH*Conc*CINN-Cl]	–0.012	0.8579

a No DoF, ordinary tests are uncomputable.

Although the mathematical model was built without any information regarding the associated errors, it is still possible to analyze the parameters’ coefficients and evaluate the factors’ importance. First of all, the coefficient associated with [NaOH] is a positive value, and it is also the highest one, indicating that the quantity of base has a direct proportional correlation with starch DS; in other words, the amount of NaOH represents a fundamental parameter to ensure high DS; in particular, given the value positivity, a high quantity of base should translate into better general outcomes. The coefficients of cinnamyl chloride and starch concentration affect DS to a lesser extent. Contrary to expectations, the [CINN-Cl] coefficient is negative, which means that starch DS is inversely proportional to the reactant concentration. Considering the previous analysis of the reaction system, it is advisable that the type of reagents and conditions used should not inhibit possible side reactions in which the cinnamyl halides can participate. Therefore, the obtained coefficient could indicate that some parasite processes exist, preventing the desired reaction. Nevertheless, in absolute values, the coefficient does not represent a real threat to the model response. Regarding starch concentration, as for the base, a positive correlation is observed: the higher the concentration, the higher the DS obtained. Regarding the interaction among factors, they can be divided into two categories: the ones with an extremely low coefficient value and the other two, which are similar to the [CINN-Cl] and [Conc] factors. In the first case (−0.011, [Conc*CINN-Cl]; −0.012, [NaOH*Conc*CINN-Cl], Table 2), the values are both negative, but due to the low amount, the effects are almost negligible in the model’s general trend. On the other hand, the other two (+0.049, [NaOH*Conc]; −0.042, [NaOH*CINN-Cl], Table 2) present some significant impacts that are easily visualizable by the contour plots (Figure S3). A positive sign of the coefficient for [NaOH*Conc] denotes that there exists a direct relationship between the two factors, reinforcing each other and affording a positive effect on starch DS. Higher concentrations of both NaOH and CINN-Cl lead to a higher DS. Conversely, the negative coefficient for [NaOH*CINN-Cl] indicates that these two factors have an inverse relationship. Moreover, in the graphs containing NaOH as a factor, it is possible to notice that the described dissected function seems to have a parabolic trend, leading to the assumption that, probably under this parameter, some quadratic terms are hidden in the data. This is in agreement with the theoretical analysis of the reaction system. In summary, the results of the SP1-DoE are the following:

(i) the base, among the studied factors, is the most relevant factor influencing the output (in agreement with the theoretical analysis of the reaction system, Section 3.2).
(ii) higher amount of NaOH and higher starch concentration promote the functionalization reaction.
(iii) the model, as evidenced in the contour plots, suggest the presence of quadratic terms, which are needed to better describe the role of the base on the DS.
(iv) low quantities of alkylating agent counterintuitively favors higher DS.

Undoubtedly, this last parameter hides some fundamental information about the reaction system. It possibly relates to side reactions between the alkylating agent and the base, which lead inevitably to byproducts; if this is the case, the addition methods of reagents to the reaction media might positively affect their interactions and the final outcome. After analyzing the results obtained through SP1-DoE, it was possible to identify some aspects of the studied reaction, which can be translated into “standard” practical conditions for the definition of a reliable procedure. First of all, the base was confirmed as a fundamental parameter as its quantities highly affect the final DS; the more it is present, the higher will be the final substitution. Between a diluted and a concentrated system, the concentration of 60 g·L^–1^ resulted to be the optimal choice. Finally, the negative coefficient of cinnamyl chloride represents the most counterintuitive outcome of the model. Lower quantities of the alkylating agent are theoretically preferred, but since the absolute value of the coefficient is not elevated, even if quantities ≥ 1.0 equiv were used, the general trend should not be drastically affected. Therefore, taking these points as starting points, it is possible to further evaluate the reaction by performing additional DoEs to explore other factors that could significantly impact the system. As a consequence, new factors were defined following the suggestions of the SP1-DoE, such as the reaction temperature during the deprotonation step, the different addition methodologies, and the type of alkylating agent, shifting from the chloride derivative to another halide. The second one was chosen as a hypothetical translation of the negative cinnamyl chloride coefficient; a portioned addition of the reagent could reduce the eventual side reactions and promote general outcomes. Increasing the temperature during the basic treatment could instead help the base penetrate inside the starch structure and afford better global deprotonation of the polysaccharide. Meanwhile, the different cinnamyl halides could improve the DS by shifting toward a reagent whose leaving group is better than the chloride moiety, such as the bromide.

#### 3.3.2 SP2-DoE–(NaOH/CINN-X­(Quantity)/CINN-X­(Type))

A more comprehensive screening of the factors was performed by evaluating how differences in the leaving group and intrinsic properties of the alkylating agents affect the reaction. In the next experimental design, information derived from the SP1-DoE FFD data was incorporated, excluding the starch concentration. Thus, the JMP software was set up for a 2-level 3-factor FFD (2^3^ FFD) with two continuous and one categorical factor reported in Table S4, which was then codified as reported in Table S5. All the experiments were performed in a randomized order using the screening reaction conditions as indicated in the Experimental Section (starch-AGU: 3.08 mmol, starch conc: 60 g·L^–1^, temp: RT; Section 2.2), except for the factors that were studied in the model (Table 3).

**Table 3:** FFD Matrix with the Outputs^a^

entry	NaOH (equiv)	CINN-X (equiv)	CINN-X (type)	DS^b^
1	1.0 [−1]	1.0 [−1]	Cl	0.01
2	3.0 [+1]	1.0 [−1]	Cl	0.55
3	1.0 [−1]	3.0 [+1]	Br	0.10
4	1.0 [−1]	1.0 [−1]	Br	0.05
5	1.0 [−1]	3.0 [+1]	Cl	0.02
6	3.0 [+1]	1.0 [−1]	Br	0.21
7	3.0 [+1]	3.0 [+1]	Cl	0.34
8	3.0 [+1]	3.0 [+1]	Br	0.18

a The corresponding coded values are reported in the square brackets.

b Determined by ^1^H NMR.

The model’s equation will be constituted by 8 coefficients including the intercept, which are schematically reported in Table 4.

**Table 4:** Estimated Effects of FFD

factors	coeff.	pseudo *p*-value^a^
intercept	0.184	
[NaOH]	0.138	0.123
[CINN-X]_(quant)_	–0.023	0.722
[CINN-X]_(type)_	0.048 (Cl)	0.485
	–0.048 (Br)	
[NaOH*CINN-X_(quant)_]	–0.039	0.565
[NaOH*CINN-X_(type)_]	0.078 (Cl)	0.294
	–0.078 (Br)	
[CINN-X_(quant)_*CINN-X_(type)_]	–0.028 (Cl)	0.674
	0.028 (Br)	
[NaOH*CINN-X_(quant)_*CINN-X_(type)_]	–0.015 (Cl)	0.813
	0.015 (Br)	

a No DoF, ordinary tests are uncomputable.

The base coefficient exhibited the highest positive value (+0.138, Table 4), consistent with the findings from the SP1-DoE, thereby emphasizing the importance of NaOH within the reaction system. Additionally, the coefficient associated with the quantity of the alkylating agent aligns perfectly with the previously reported data (Section 3.3.1), further validating the model. The coefficient corresponding to the reagent type was positive (+0.048) for cinnamyl chloride, in contrast to bromide (−0.048), indicating that a higher DS outcome is obtained using chloride. This behavior is further underlined by the interaction factor of the halide with the base ([NaOH*CINN-X_(type)_] = ± 0.078), which indicates a preference for chloride over bromide. An intriguing interaction effect between the type and the quantity of the alkylating agent (±0.028) is observed: the coefficient is positive for bromide and negative for chloride.

These findings suggest that, at higher equiv of alkylating agent, bromide is preferred, whereas at lower equiv, chloride prevails. This behavior is consistent with the SP1-DoE discussed in Section 3.3.1. Furthermore, the different patterns of the two alkylating agents are also evident from the experimental DSs obtained with various base:halide ratios (as illustrated in Figure 2); in this context, the two alkylating agents behave oppositely, depending on the chosen reaction conditions. Cinnamyl chloride yields the best results at high equivalents of base, especially when CINN-X quantities are limited, corroborating the initial screening observations. Conversely, at low equivalents of base, the functionalization is extremely limited for both halides; nevertheless, bromide demonstrates superior performance (Figure 2). Overall, it can be affirmed that higher quantities of base enhance the reactivity of both halides. This effect is inversely proportional to their quantity, with cinnamyl chloride exhibiting better performance under these conditions. On the other hand, with a low amount of base, bromide emerges as the more effective choice, and its effectiveness is directly proportional to its quantity.

**Figure 2:**
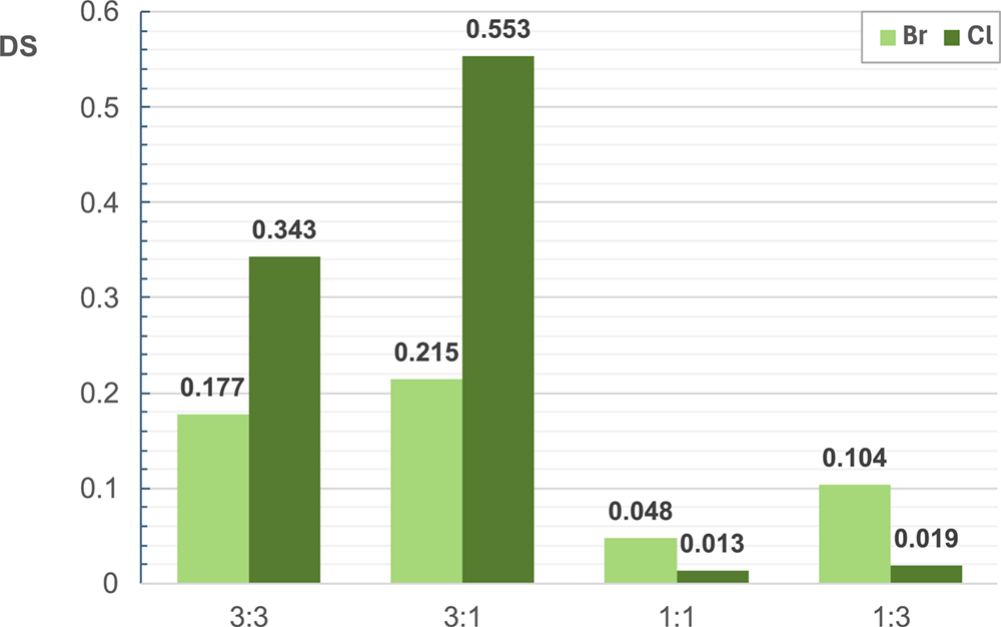
Bromide and chloride DS comparison at different NaOH:CINN-X ratios.

These discrepancies may be ascribed to their reactivity characteristics. Cinnamyl bromide, due to the intrinsic presence of bromine as a better leaving group, presents a higher reactivity toward nucleophilic substitution when compared to chloride. Nevertheless, this reactivity advantage is lost when more equivalents of base are used, probably because of its reactivity, which makes it more susceptible to side reactions. Conversely, when the base is limited, the bromide’s higher reactivity compensates, promoting the functionalization and providing better results than its chlorinated counterpart.

In summary, the results of SP2-DoE are the following:

(i) the base, among the studied factors, is the most relevant factor influencing the output (in agreement with the previous screening, SP1-DoE)
(ii) the effectiveness of cinnamyl halide is dependent on the reaction conditions:
  (a) chloride is preferred at higher base equivalents
  (b) bromide is preferred at lower base equivalents
  (c) chloride is more prone to side reactions at higher equivalents, thereby sacrificing starch functionalization

#### 3.3.3 SP3-DoE–(Temperature/Addition)

An FFD approach was used to screen the effect of temperature on alkoxide formation and the alkylating agent addition methodology as the factors, with no replicates. Thus, the JMP software was set up for a 2-level, 2-factor FFD (2^2^ FFD) with continuous (temperature) and categorical (addition methodology) factors (Table S6). The continuous factor was coded with ±1 values; meanwhile, the categorical factor was coded with “L1” and “L2” as reported in Table S7. All the experiments were performed in a randomized order using 3.0 equiv of NaOH, 1.0 equiv of CINN-Cl, and a starch concentration of 60 g·L^–1^ as reaction conditions. The multiple additions of cinnamyl chloride have been made in three equal portions every 2 h. The collected data are reported in Table 5.

**Table 5:** FFD Matrix with the Outputs^a^

entry	temp (°C)	addition	DS^b^
1	RT [−1]	portion-wise [L2]	1.19
2	RT [−1]	one-shot [L1]	0.55
3	90 [+1]	one-shot [L1]	0.30
4	90 [+1]	portion-wise [L2]	1.55

a The corresponding coded values are reported In the square brackets.

b determined by ^1^H NMR.

As highlighted in Table 6, the coefficients associated to both [Addition] and [Temp*Addition] factors exhibit different signs, which is due to the presence of a categorical one. The latter directly dictates the sign of the coefficients as a function of the type of addition employed; in particular, positive signs are linked to portion-wise additions, whereas negative ones correspond to the one-shot methodology. Specifically, talking about the coefficients, they result in all negative values when one-shot addition is used (Table 6), indicating a detrimental impact on the overall DS. In contrast, the addition of multiple portions yields positive coefficients, which positively affect the DS. Moreover, the addition method resulted in being particularly important since the related coefficient is much larger than the others, suggesting that the mode of reagent introduction exerts a substantial impact on the process. Regarding the temperature, its coefficient is low and negative, indicating that an increase in temperature will not lead to an improved overall outcome. Even the interaction factor [Temp*Addition] is influenced by the sign of its coefficient; specifically, the addition method actively discriminates between direct and inverse relationships among the factors. When portionwise additions are employed, the interaction indicates a direct proportionality, implying that an increase in temperature could potentially enhance the overall process performance. Temperature is known to be an important factor in promoting chemical reactions (and side reactions); however, the model suggests that the temperature has a limited effect. Based on these observations, starch functionalization with cinnamyl moieties can be conducted at room temperature with good results, a condition that is particularly advantageous considering potential industrial applications.

**Table 6:** Estimated Effects of FFD

factors	coeff.	pseudo *p*-value^a^
intercept	0.899	
[temp]	–0.0258	0.9279
[addition]	0.4711 (portion-wise)	0.2858
	–0.4711 (one-shot)	
[temp*addition]	0.1513 (portion-wise)	0.6257
	–0.1513 (one-shot)	

a No DoF, ordinary tests are uncomputable.

In summary, the results of SP3-DoE are the following:

(i) the addition mode of the alkyl halide is a key factor: the portion-wise methodology is by far the most effective procedure
(ii) temperature has a poor influence on the process: room temperature is used as an operational condition in the following steps.

### 3.4 Base Variation

Based on the screening phase results, which highlighted the base as the major effector in the reaction system and on some experimental observations, a deeper study was envisaged.

Among the various problems encountered during the experiments, the most significant involved the base itself, alongside issues with starch gelatinization and high mixture viscosity. We initially hand-crushed the base (NaOH) into a fine powder before adding it to the reaction batch. However, sodium hydroxide is known to poorly dissolve in DMSO, particularly at room temperature in a highly viscous solution. Consequently, instead of a homogeneous solution, we ended up with a dispersion of small solid fragments of NaOH floating in the mixture. This was a significant issue because a base dispersion, as opposed to a solution, drastically reduces the surface interaction between the base and starch substrate. This also meant that the reaction operated as a heterogeneous system, which further complicated the process. The nonoptimal NaOH solubility may also favor side-reactions (i.e., nucleophilic attack on the alkyl halide) instead of the desired starch alkoxide formation. In particular, the nucleophilic behavior of NaOH was proved since cinnamyl alcohol (CINN-OH, *R*_f_: 0.62 - PE:EtOAc 6:4, Figure S8, Supporting Information) was identified in every single test performed by TLC, and isolated, and characterized as a reaction byproduct in some of them, alongside the dicinnamyl ether (CINN-O-CINN, Figure S9, Supporting Information) achieved by further consumption of NaOH and CINN-X. These observations also provide support to the screening results: since the NaOH is actually consumed in other by-processes leading to the formation of CINN-OH, higher quantities of base are consequently needed to ensure higher DS. In addition, the interaction between NaOH and CINN-Cl emerging from the screening phase, which may negatively affect deprotonation efficiency due to the heterogeneous conditions, could also influence the CINN-Cl coefficient observed in the statistical model.

To better understand the base role in the reaction system, NaOH was substituted with different alkali, which are perfectly soluble in DMSO. As a first alternative, the NaDMSO (sodium dimsyl) ion was evaluated since it is produced from neat DMSO and NaH (sodium hydride) (Scheme S2, Section S1.1.1); it is a stronger base than sodium hydroxide, and its use is already reported in the literature^26,27^ for polysaccharide functionalization.

Dimsyl anions are known to be extremely reactive (p*K*_a_ ≈ 36) and unstable, especially toward water, oxygen, and carbon dioxide, which requires their usage in a very strict inert and controlled environment. Indeed, if traces of water are present, the anion would react generating NaOH that can undergo the same side-reactions previously reported. However, its strong basicity is expected to prevent a nucleophilic behavior, facilitating the deprotonation rather than nucleophilic substitution.

Sodium alkoxides were also considered as interesting alternatives since they are soluble in DMSO, and the solvent itself enhances their basicity through coordination with the sodium ions, freeing the negative counterions. Specifically, sodium alkoxides derived from methanol, ethanol, and *iso*propanol (NaOMe, NaOEt, NaO*i*-Pr) were prepared (Section S1.1.3) and tested.

These organic bases were chosen due to their properties and to additionally evaluate how decreasing nucleophilicity from methoxide to *iso*propoxide would affect the deprotonation step and the whole process (Scheme 3). As steric hindrance increases around the alkoxide group, nucleophilicity decreases; this phenomenon then prevents hindered alkoxides from participating as nucleophiles and affects the final outcome. In general, it is expected that sodium methoxide would provide worser DS yields when compared to ethoxide and especially *iso*propoxide.

**Scheme 3:**
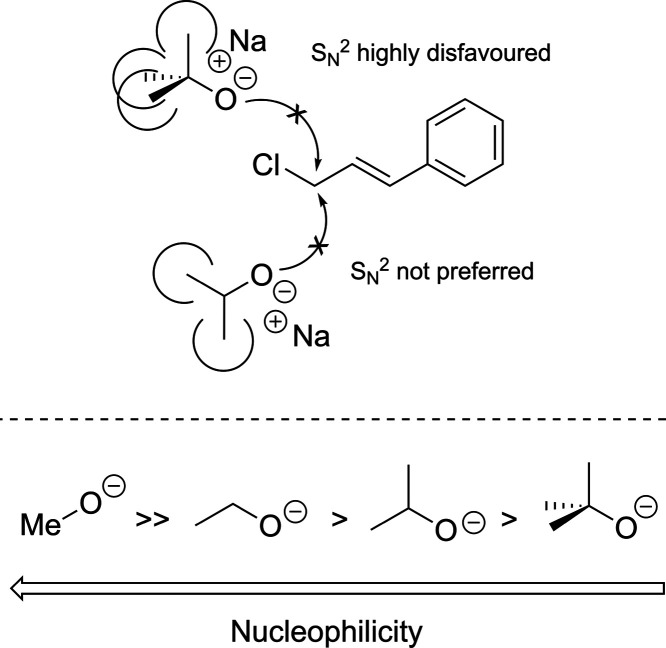
Schematic Representation of How Steric Hindrance Disfavors the Nucleophilic Substitution (Upper Part of the Scheme)^a^

a Sodium alkoxides are arranged following their reactivity from primary to tertiary, respectively, methoxide, ethoxide, *iso*propoxide, and *tert*-butoxide (in the lower part).

Nevertheless, even sodium alkoxides are extremely sensitive to moisture; moreover, they may promote additional side reactions since it is known that some of them might act as initiators in anionic polymerizations of olefins. Furthermore, the alkoxides could also act as nucleophiles, forming an ether with the cinnamyl halide; however, this would not interfere with the rest of the reaction, since this would not be further deprotonated or attacked by other nucleophiles, unlike the reaction involving NaOH.

Going back to NaOH, it was not completely discarded as a base; instead, a fine dispersion in DMSO (Supporting Information, Section S1.1.2) was prepared before addition to the reaction media.

As reported in Figure 3, the alternative bases follow the general trend observed for the standard procedure with NaOH, providing a similar DS and even better outcomes. It is important to underline that any different base that has been used enhances the gelification issue after the addition, hampering practical and large-scale application.

**Figure 3:**
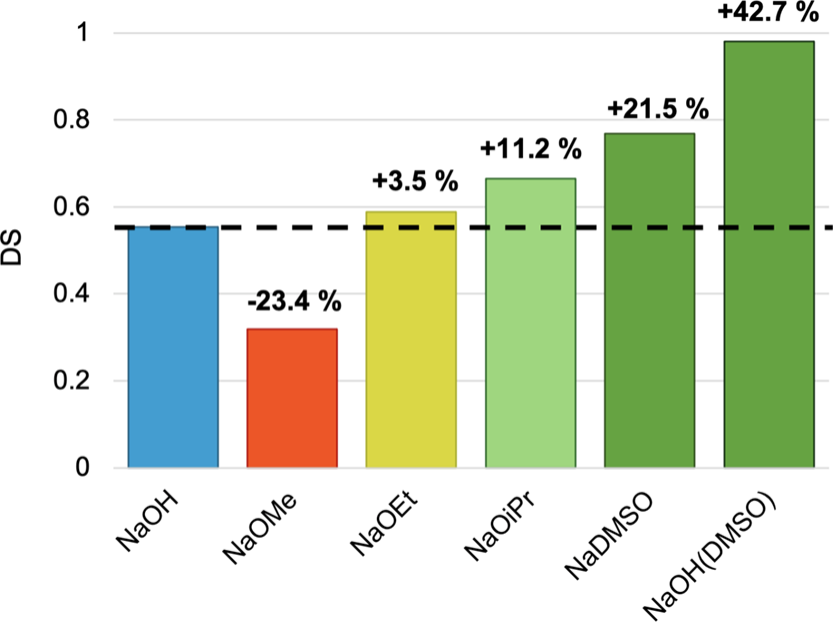
Percentual DS gain and loss using different bases calculated as *E*_DS_ – *E*_DS_^0^, where “*E*_DS_” and “*E*_DS_^0^” are the percentage effectiveness of the experiments and the control reaction, respectively, and *E*_DS_ = (DS_exp_/DS_theo_) × 100.

Sodium alkoxides followed the trend corresponding to their respective steric hindrance effect (from −23.4% to +11.2% of DS, Scheme 3). Notably, NaOMe likely suffered limitations attributable to its intrinsic nucleophilicity. In contrast, NaOEt and NaO*i*-Pr demonstrated that increased steric bulkiness could effectively mitigate side reactions, resulting in DS values comparable to or even exceeding those of the reference case (Table S1). As illustrated in Figure 3, sodium dimsyl ions resulted in an increase of 21.5% in DS relative to the control reaction; however, the complex preparation, handling, and storage requirements, alongside the reagents necessary for its synthesis, significantly constrain its practical applicability, particularly in the context of potential industrial scalability. Finally, the most favorable outcome was achieved with NaOH dispersed in DMSO (+42.7%, Figure 3), almost reaching the theoretical value of DS (Table S1). This, alongside the good solubility of all other tested bases, suggests that the heterogeneity in the control reaction significantly hampers the reaction, thereby limiting its effectiveness and demonstrating the potential of these alkalis as valid alternatives.

### 3.5 Optimization Phase–Central Composite Design

The screening phase of several reaction parameters through prior DoE methodologies afforded indicative data that laid the basis for the optimization phase. To perform this, a central composite design (CCD) was selected, considering the number of factors to be evaluated. The CCD can be regarded as a refined extension of an FFD, achieved through the incorporation of a star design (SD), as illustrated in Figure 1. By expanding the experimental space of a classical FFD, the CCD introduces additional points (±α, namely, axial points) located outside the conventional factorial domain, effectively delineating a sphere, or circumference, encompassing the original cube, or square, of the FFD. This approach allows for a more comprehensive exploration of the experimental space and better modeling of the response surface. The inclusion of these external points in the experimental design may further challenge the methodology, as they could correspond to extreme practical conditions that are difficult to replicate in a real-world application. Unlike previous models, this analysis utilizes a mathematical equation characterized by six coefficients, including the intercept (eq S5, Section S1.4.2). Additionally, five central point replicates were performed, rather than the usual three as suggested by the software, to better assess the statistical significance of the estimated parameters, the model error, and the general robustness of the prediction.

Given that temperature, starch concentration, cinnamyl bromide, sodium alkoxides, and sodium dimsyl ions did not appear significant to DS maximization, the quantities of NaOH and cinnamyl chloride were considered as primary factors under study. Based on the screening experiments, the procedure was modified to incorporate the use of finely dispersed NaOH in DMSO and the portion-wise addition of the alkylating agent. These modifications were implemented, as they led to a significant increase in the final DS of the starch. The JMP software is configured to perform a 2-level 2-factor CCD, with both factors modeled as continuous variables (Tables S8 and S9).

In the majority of the optimization experiments, the formation of two distinct solids was observed. They exhibited differences in terms of their DS, solubility in organic solvents (DMSO and chloroform), and their precipitation pathway during the reaction workup, with one solid forming during the initial precipitation and the other precipitating from the mother liquor obtained afterward; for sake of clarity, based on this the solids will be designated as “solid a”, “solid b”, respectively. Following this observation, the outputs considered in the optimization phase are the DSs and the weights of both solids.

Table 7 presents the complete experimental matrix alongside the corresponding responses data. The analysis of each output is analyzed individually and optimized independently. In particular, the model is configured to maximize all responses except for the weight of “solid a”. This exception is due to the fact that when “solid a” is obtained concomitantly with “solid b”, it typically exhibits pronounced insolubility in both aqueous and organic solvents, thereby significantly constraining its potential for further applications.

**Table 7:** CCD Matrix with the Outputs^a^

			DS^b^		weight (mg)	
entry	NaOH (equiv)	CINN-X (equiv)	a	b	a	b
1	2.0 [0]	0.586 [−1.414]	0.54	na	796.4	0
2	2.0 [0]	2.0 [0]	1.48	1.62	207.2	471.9
3	2.0 [0]	2.0 [0]	0.90	1.82	167.3	518.9
4	1.0 [−1]	1.0 [−1]	0.36	1.17	487.4	137.6
5	2.0 [0]	2.0 [0]	1.60	2.07	144.0	589.8
6	2.0 [0]	3.414 [+1.414]	1.44	1.98	185.7	523.0
7	2.0 [0]	2.0 [0]	1.33	1.93	170.5	536.2
8	3.414 [+1.414]	2.0 [0]	1.07	1.33	399.8	420.1
9	0.586 [−1.414]	2.0 [0]	0.13	0.97	525.9	57.0
10	3.0 [+1]	1.0 [−1]	0.49	na	877.0	0
11	1.0 [−1]	3.0 [+1]	0.35	1.33	423.0	233.0
12	3.0 [+1]	3.0 [+1]	1.63	2.02	274.5	569.4
13	2.0 [0]	2.0 [0]	1.41	1.78	164.8	569.9

a The corresponding coded values are reported in the square brackets.

b Determined by ^1^H NMR/na: not available.

#### 3.5.1 DS of “Solid a”

The data, illustrated in Figure 4A, indicate that all five investigated factors exhibit statistically significant effects on the output (source data are reported in Table S10, Supporting Information). The final DS is predominantly governed by the NaOH quantity and its quadratic term (+0.3431 and −0.3933). Additionally, the positive interaction factor coefficient (+0.2858, [NaOH*CINN]) corroborates a direct relationship between the base and the cinnamyl reagents. Conversely, the quadratic terms referred to as reagent equivalents ([NaOH]^2^ and [CINN]^2^) exert a specific influence on the response surface curvature due to the negative sign, causing a maximum in the response surface, effectively constraining an upper limit of achievable DS within a specific range of values (Figure 5A,B). Overall, the observed trend remains consistent with the screening findings (SP1–3-DoE).

**Figure 4:**
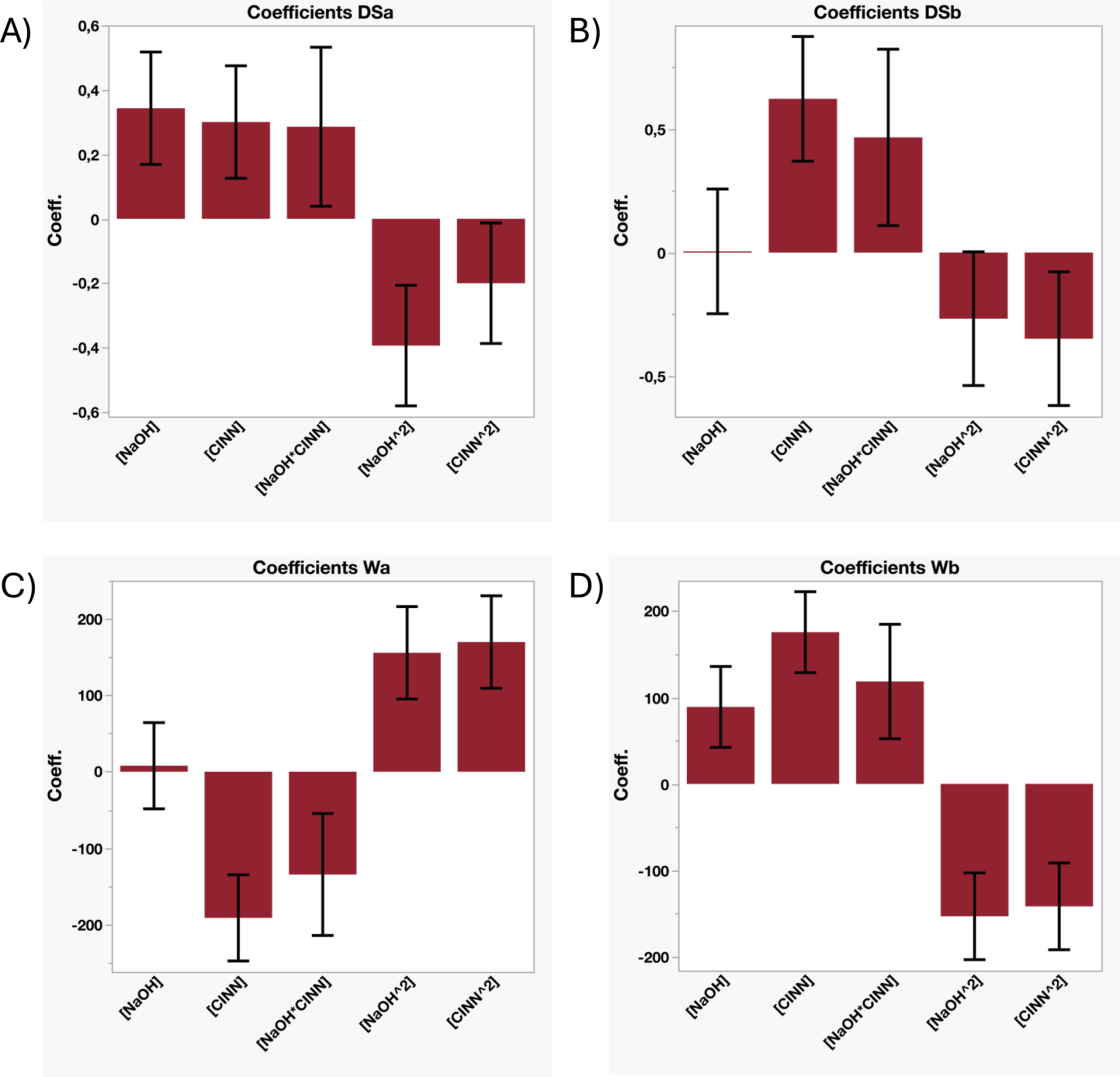
Response coefficients with the confidence intervals (indicated in black). Results whose confidence interval crosses zero-line (no effect) do not achieve statistical significance.

**Figure 5:**
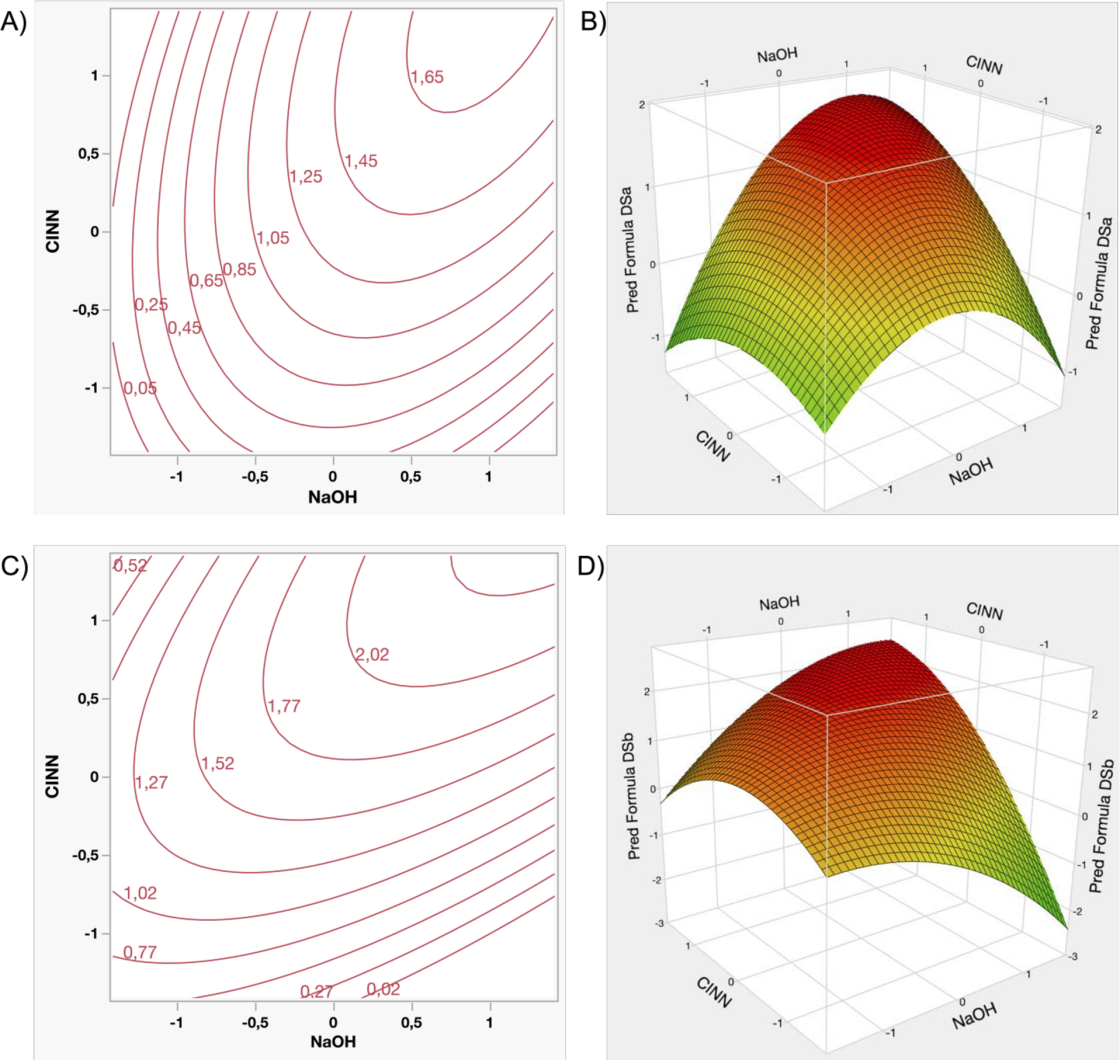
Contour and Response Surface Plots of “DSa” (A–B), and “DSb” (C–D).

The corresponding *R* ^2^ is 0.91, indicating a high level of model fit and demonstrating the capacity to adequately describe the observed experimental trends. However, the proportion of explained variance by the model is limited to 85.2%, which, while acceptable, suggests that approximately 14.8% of the variability remains unaccounted for. This residual variability may be attributable to unconsidered factors or a more complex underlying reaction system.

#### 3.5.2 DS of “Solid b”

The effects of the factors on the output differ markedly from those previously observed, as illustrated in Figure 4B (source data are reported in Table S11, Supporting Information). Notably, for the first time, the quantity of NaOH does not exhibit statistical significance; also, its quadratic term exerts a limited influence within the model. Conversely, the amount of cinnamyl chloride significantly impacts the maximum achievable DS (Figure 5C,D). The proportional relationship between the two factors remains consistent, and the quadratic terms contribute to the establishment of an upper limit for the DS, indicating a response surface maximum. *R* ^2^ is reported as 0.89, corresponding to a total explained variance of 81.1%. While these values suggest that the model reasonably captures the experimental trend, some residual variability remains unaccounted for, implying again that additional factors or more complex processes may influence the reaction system (vide infra Section 4), beyond those included in the current model.

#### 3.5.3 Weight of “Solid a”

The estimated effects of each factor are resumed in Figure 4C (and in Table S12, Supporting Information). The mathematical model clearly shows that complete elimination of “solid a” appears unfeasible. In fact, although NaOH does not demonstrate statistically significant effects, its presence is nonetheless essential for the successful progression of the reaction, ultimately leading to the formation of “solid a”. Its quadratic term, alongside with the one related to the cinnamyl chloride, imposes a limit on minimization (Figure 6A-B), resulting in a defined lower bound for the obtainable quantity of “solid a”. Aside from that, particular emphasis on the amount of cinnamyl chloride, and its interaction factor [NaOH*CINN] appears. The model yields an *R* ^2^ value of 0.96, with a total explained variance of 92.5%. Compared with the other responses, these values are markedly higher, indicating that the model provides a more accurate representation of the factors governing the formation of “solid a”.

**Figure 6:**
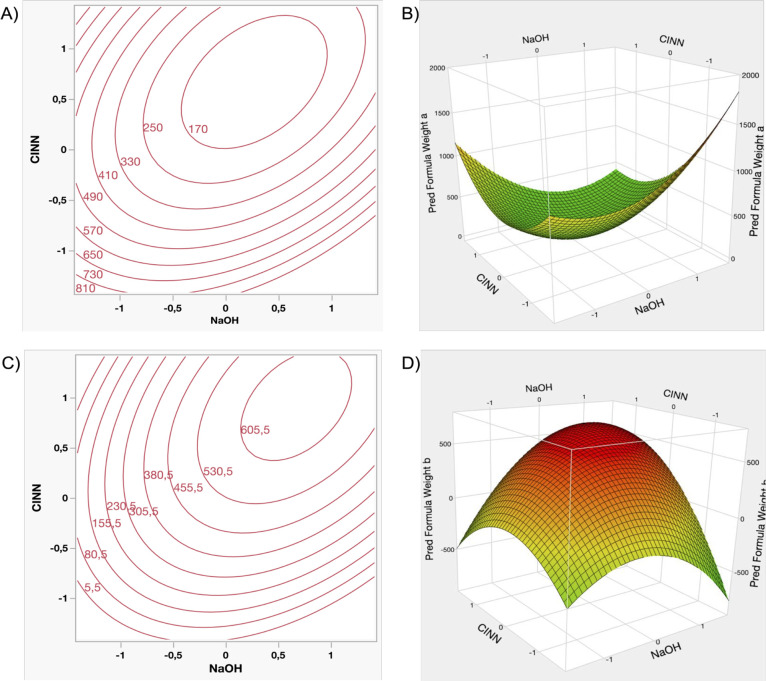
Contour and Response Surface Plots of “Weight a” (A, B), and “Weight b” (C, D).

#### 3.5.4 Weight of “Solid b”

The data (Table S13, Supporting Information) indicate that all factors exert statistically significant effects (Figure 4D), with signs that are entirely opposite to those observed in the immediately previous analysis. While the formation of “solid a” was previously statistically unaffected by the quantity of base used, the formation of “solid b” is now significantly influenced by both reagent quantities. Figure 6C,D illustrates the corresponding response surface and contour plots, which visually represent the numeric interaction of the several factors. The model yields an *R* ^2^ value of 0.97, with an explained variance of 94.3%. These values are among the highest obtained throughout the analysis, suggesting that at least for the formation of both solids, the constructed model reliably captures the underlying trend with high accuracy.

To summarize the overall results of the proposed detailed study of starch functionalization through ether linkage to cinnamyl moieties, the optimization phase outlined the following key information:

(i) base and alkylating agent equivalents are key variables toward optimization of starch functionalization
(ii) complex and competing side reactions do occur, depending on the nature of the alkylating agent and its ratio to the base equivalents
(iii) the reaction system shows a nonlinear behavior in respect to the most relevant factors, that is, the halide and the base equivalents used (quadratic effects are present in the model)
(iv) the experimental space studied affords two “solids” as recovered products, which differ in terms of DS, solubility, and amount as a function of the specific experimental conditions
(v) it is impossible to selectively obtain “solid b” without “solid a”; however, the reverse is possible
(vi) a maximum DS of around ≈ 2.5 can be reached in the optimal conditions, corresponding to the experimental upper limit
(vii) the model resulted to be robust to comprehensively describe the selected experimental space

## 4 Reaction System Rationalization

Thanks to the extensive and comprehensive DoE statistical study approach, we were able to further explore the discussed reactive system and consequently present our hypotheses regarding the principal side reactions that may occur and potentially negatively affect the reaction outcomes, drawing from experimental evidence gathered over several months of work (Figure S23).

As previously described in Section 3.4, the bases employed, particularly the most nucleophilic ones, promote the formation of various byproducts, such as cinnamyl alcohol and dicinnamyl ether, leading to significant reagent consumption. Furthermore, our findings indicate that the basic treatment of the bare starch likely results in a situation where a portion of the introduced NaOH is consumed by other processes, which is also related to the disruption of the polysaccharide’s micro- and macrostructure (Section S6).^28^

In addition to these points, we observed that the precipitation of the cinnamylated starch was compromised by the choice of the solvent. Acetone proved unsuitable for this purpose, as we have reason to believe that highly basic environment strongly promotes the self-condensation reactions, leading to the formation of a spectrum of small molecules, such as mesityl oxide, phorone, and isophorone via Michael addition, and/or even more complex structures.^29–31^ These compounds exhibit a characteristic yellow-red coloration, which is one of the most consistently observed colors in the final functionalized starch after the precipitation step. However, their identification in the final products through NMR analyses was challenging, partly due to the broad background signal from the starch which possibly overlaps other small sharp signals.

Further focusing on the reaction conditions, these may promote an oxidative environment; indeed, the recurrent detection of the dimethyl sulfide odor supports this possibility. Consequently, it is plausible that within the main batch, certain oxidative side reactions, such as the Kornblum reaction,^32^ may proceed concurrently with the desired process, yielding various oxidized products, notably cinnamaldehyde. Although this specific molecule was never detected by NMR, it could have participated in other rapid secondary reactions. For instance, under the chosen experimental conditions, aldehyde may have been oxidized to cinnamic acid and/or involved in Corey–Chaykovsky reactions,^32^ leading to the formation of characteristic oxiranes. Furthermore, given the strongly basic environment, cinnamaldehyde can also undergo retro-aldol reactions to yield benzaldehyde and formaldehyde, which would make them subject to further oxidation, self- and aldol condensation, and disproportional processes. We found it particularly intriguing that the latter two aldehydes (benzaldehyde and formaldehyde) might eventually react with the non-reducing ends (NRE) of the starch to form acetals, occupying the C4 and C6 positions (the ones naturally present in the granule, and the ones that might be formed after the basic degradation^28,33^ ). This behavior is subtle, because it is not readily detectable by analyzing only the cinnamic portion of the NMR spectra. Nevertheless, a broad signal is consistently present in the functionalized starch NMR spectra at approximately 5.6 ppm in DMSO-*d*_6_, as it can be noticed in Figures 7 and S11–S19.

**Figure 7:**
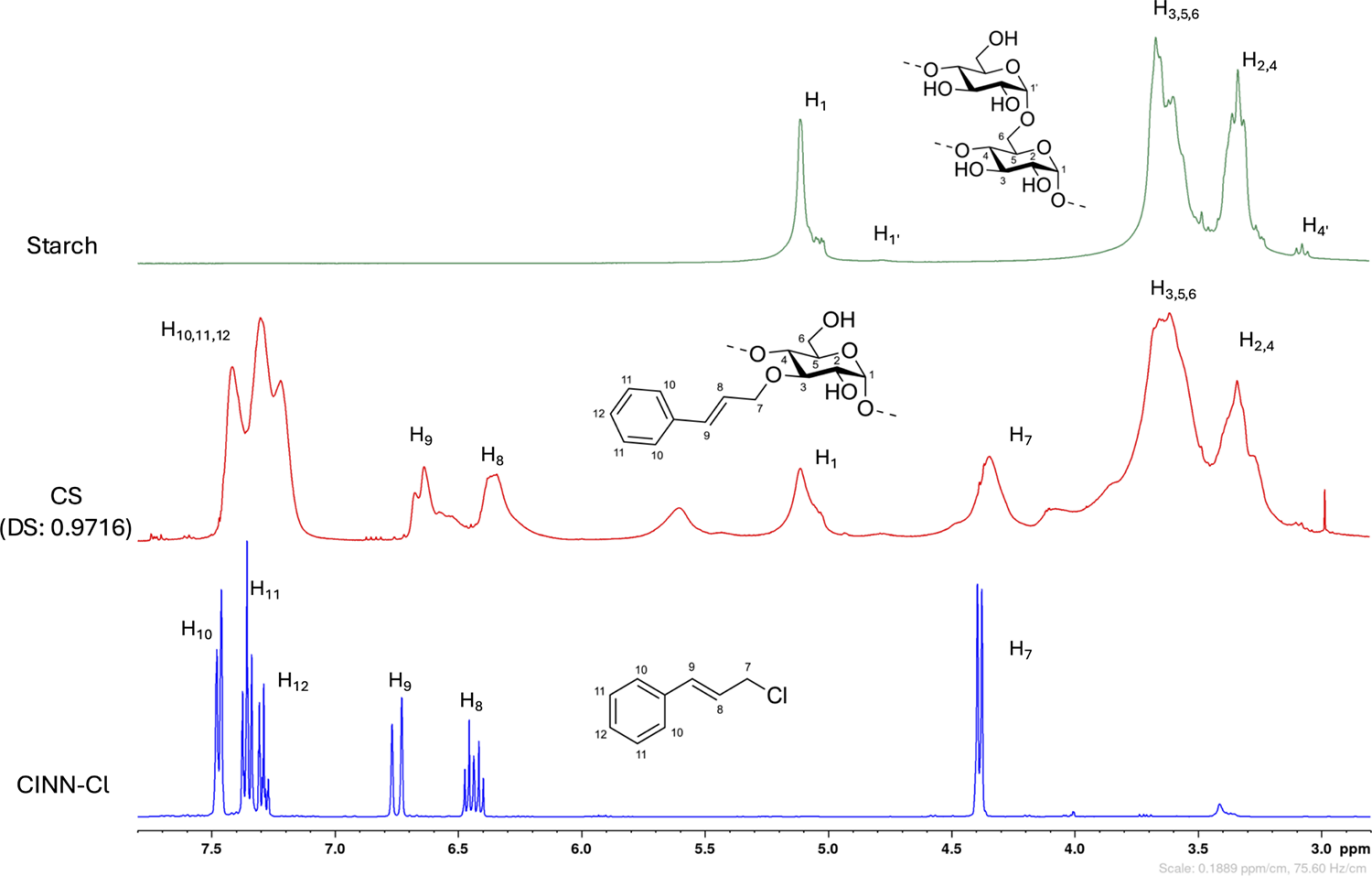
Comparison of ^1^H NMR in DMSO-*d*_6_ of pristine starch (green), cinnamylated starch (namely, CS, red), and cinnamyl chloride. Starch and CS samples were recorded with the addition of TFA-*d*. The numbering was assigned arbitrarily. ^1^H NMR CINN-Cl (DMSO-*d*_6_, 400 MHz) δ [ppm]: 7.48–7.36 (m, 2 H), 7.36–7.34 (m, 2 H), 7.31–7.27 (m, 1 H), 6.75 (d, *J* = 15.8 Hz, 1 H), 6.44 (dt, *J* = 15.7, 7.22 Hz, 1 H), 4.39 (d, *J* = 7.22 Hz, 2 H).

This hypothetical assignment has been historically complicated; however, considering acetal formation, this signal might constitute direct evidence of the process, as suggested in the literature.^34^ To further elucidate this hypothesis, we performed a comprehensive NMR characterization on a highly substituted cinnamylated starch, anticipating detectable differentiation of chemical shifts among the various acetalic signals. Regrettably, ^1^H-^1^H COSY (correlation spectroscopy) and ^1^H-^13^C HSQC (heteronuclear single quantum coherence) experiments did not provide direct evidence for a correlation between this unusual signal and the cinnamic moiety. Nonetheless, in the ^13^C­{^1^H}-APT (attached proton test) NMR, its chemical shift and peak phase established that it corresponds to a CH group, placing it extremely close to the acetalic starch-AGU carbon spectrum zone. Consequently, we achieved neither direct proof of the existence of the additional acetalic moiety nor disproved our hypothesis. A dedicated HMBC analysis could be beneficial for further investigation, particularly to distinguish between the possible acetals formed from cinnamaldehyde and benzaldehyde, respectively. Should the outcomes be positive, understanding the acetal formation mechanism under highly basic conditions, an environment known to impede this type of process, would also be of extreme interest, although completely being beyond the goals of the present work.

Finally, regarding the formation of two distinct solid fractions during the workup, additional FT-IR analyses indicated that the insoluble solid might be primarily constituted by a high percentage of inorganic reaction byproducts, alongside a small fraction of functionalized starch that coprecipitated early in the workup step. This would justify the observed DS values determined by NMR for this fraction, despite its drastic insolubility. Specifically, where the percentage of cinnamylated starch is extremely low, the discrepancy between DS and sample weight is significant, possibly compounded by low spectral resolution; conversely, when the fraction is slightly higher, the DS values and weights show greater similarity.

## 5 Conclusions

This study offers a comprehensive investigation into the functionalization of starch, employing the DoE statistical approach for both screening and optimization. The work was built upon a methodology previously developed by our research group^17^ for synthesizing functionalized starch intended for use in UV-sensitive, biobased materials. The resulting data provide clear insights into competing reactions, which in turn suggest strategies for mitigation and optimization. To the best of our knowledge, this represents the first detailed inspection of a reaction system focused on starch functionalization by cinnamyl halides via base-mediated etherification.

### 5.1 Strengths of the Approach

The reaction pathway was initially explored at a theoretical level: several reaction parameters were included in the screening phase and systematically investigated using FFDs to elucidate their influence on the process outcomes. Among these, the quantity of base and the alkylating agent addition methodology emerged as the most influential parameters. Given the critical role of the base in steering the competition between starch functionalization and competing reactions, alternative bases to NaOH were explored. Among the options tested, the use of “finely dispersed” NaOH in DMSO proved to be optimal, resulting in higher DSs. Regarding the alkylating agents, the experimental design approach provided additional crucial information that might have otherwise been overlooked. The power of the DoE over the one variable at a time (OVAT) approach clearly emerges when considering the effect of the nature of the alkyl halide on the reaction system. This effect was found to be strictly interconnected with the base equivalents; in other words, a significant second-order interaction exists and must be considered to fully describe the reaction.^19^ These insights allowed us to adopt suitable strategies, resulting in the portion-wise addition of the alkylating agent, to achieve the best DS and minimal side reactions.

Variables such as temperature and starch concentration did not yield statistically significant effects within the adopted experimental procedure; however, their examination still provided valuable insights into the reaction system.

Following the screening phase, CCD was employed for process optimization. The resulting model was statistically robust and capable of reasonably capturing the experimental trends. The latter successfully optimizes both the DSs and the weights of the two solids obtained from starch functionalization, which are distinguished by their precipitation pathway. No significant outliers were detected, and all of the data points were retained for analysis. This was further corroborated by the resulting robustness of the models, as substantiated by the obtained *R* ^2^ values.

The CCD model can be used as a predictive tool for achieving specific DS values. For instance, within the parameter ranges shown in Figure S4, it is possible to obtain DS between 1.43 and 1.65 for “solid a” and between 1.91 and 2.13 for “solid b” by employing a cinnamyl chloride range from 2.42 to 2.94 equiv, and a NaOH range from 1.97 to 2.54 equiv. These same conditions also correspond to obtaining more than 500 mg of “solid b” and less than 120 mg of “solid a”, thus demonstrating the model’s utility as a comprehensive predictive framework. To evaluate the model’s predictive capability, a validation experiment was performed at a selected “sweet spot” (Figure S21). The results for all response variables fell well within the calculated prediction intervals (PIs), confirming the model’s overall validity (Table S14, Supporting Information). This indicates that the observed discrepancies between theoretical predictions and experimental outcomes are statistically congruent with the estimated residual variance (or background noise) of the system.

The entirety of the experimental design provided a critical refinement of both theoretical understanding and experimental knowledge regarding the studied system, surpassing our initial expectations. For further details, subtle characteristics are appended in Section 4, adding a more comprehensive visualization of the system’s behavior, also expanding into isolation, workup procedures, and mechanism rationalization.

### 5.2 Limitations of the Approach

#### 5.2.1 Model Approximation

The model was designed primarily to optimize the DS; currently, the model provides no insight into product physical properties, chemical homogeneity, regiochemistry of functionalization, amylose/amylopectin content, or crystallinity. Obtaining this critical information requires further study using cutting-edge analytical techniques, given the inherent complexity of starch as a polyfunctional polymer composed of two structurally different components. Despite being developed to optimize DS, the experimental conditions afforded two solids (namely, “solid a” and “solid b”) as functionalization products, and their weights were hence considered as additional responses. Based on the generally observed unfavorable properties of “solid a” (e.g., insolubility), the model was constrained to minimize its weight response. Critically, this minimization is invalid when “solid a” is the sole product obtained, a situation in which its weight should actually be maximized. This software constraint, which requires a response to be strictly maximized or minimized, inherently limits the model’s accuracy in reflecting the full experimental reality. The CCD may therefore inaccurately model subtle reaction trends because it does not incorporate the effects of more complex mechanisms, including side reactions, starch backbone degradation, crystallinity, and the regioselectivity of OH functionalization. This oversight might be evidenced by the model’s low explained variance.

#### 5.2.2 Base Side Reactions

To further substantiate the complexity of the reaction system, a study by Chi et al.^28^ on starch reported that after only 10 min of exposure to 0.3 M NaOH aqueous solution, significant degradation of the polysaccharide backbone occurred, involving cleavage of both α-1,4 and α-1,6 linkages. We also conducted an exploratory experiment (detailed in Section S6) to determine if this phenomenon occurs in the NaOH/DMSO system. The results confirmed NaOH’s involvement as a reagent in side reactions. Such a substantial depletion would negatively impact the efficacy of the starch functionalization reaction, notably constraining the maximum achievable DS. As determined by NMR analysis, the maximum attainable DS for the potato starch employed in this work is approximately 2.964; however, even under optimal conditions, the experimental DS values did not reach the theoretical limit, underlining that the chemistry beyond starch functionalization does intrinsically impose limitations on the maximum DS attainable, as correctly foreseen by the model (i.e., ≈2.5 maximum DS for “solid b”).

#### 5.2.3 Workup Criticalities

In the reaction workup, acetone was used as a precipitation solvent. It was observed that it does not behave as an inert solvent, giving rise to a series of reactions during the precipitation step. These processes appeared to lead to the formation of various byproducts, which were detected by NMR in the final isolated solids despite multiple washing steps. Such phenomena could have implications for the DoE and the interpretation of results, further highlighting the complexity of the experimental system.

#### 5.2.4 Starch Gelatinization

One notable issue was the gelification associated with the addition of bases to the reaction mixture. The behavior of the reaction system varied depending on the amount of base used; at higher equivalents, gelification occurred almost immediately upon addition. Conversely, at lower equivalents, gelification proceeded slowly or did not occur at all. This process contributed to increased heterogeneity within the reaction batch, complicating subsequent handling and processing.

#### 5.2.5 Addition Methodology Control

Although the portion-wise addition of cinnamyl chloride was identified as one of the most effective strategies to optimize overall reaction outcomes, it was observed that only the tests employing a systematically reduced amount of cinnamyl chloride per addition, combined with an appropriate quantity of base, yielded the most favorable results and eventually the formation of just one of the two solids. Specifically, these conditions demonstrated a small or the smallest discrepancy between the experimental DS and the expected DS. This suggests that implementing an even more controlled addition protocol, such as through a syringe pump, could potentially enhance reaction efficiency by minimizing the occurrence of side reactions.

#### 5.2.6 Predictive Intervals

The optimization model demonstrated high descriptive fidelity, as supported by high *R* ^2^ values and substantial explained variance. This confirmed the model’s robust capacity to describe the main factor interactions. However, the resulting wide PIs suggested that the system’s inherent high background noise would limit the model’s overall predictive accuracy. As a fact, the validation experimental values (Table S14, Figure S22) marginally exceeded the confidence intervals (CIs) for the predicted mean, except for DSb. This outcome is not contradictory but rather a direct reflection of the large residual variability inherent to the system.

In conclusion, our study, employing rigorous screening and optimization processes, has significantly deepened our understanding of the inherent complexity governing starch functionalization. These critical findings not only substantially augment the existing body of knowledge but, more importantly, establish a novel conceptual framework. The latter is crucial, as it effectively lays the groundwork for all subsequent research and investigative endeavors in this field. Furthermore, it sets the bases for an even possible expansion toward the study of other polysaccharides, equivalent or similar to starch, leading to a new page of research.

## Supplementary Material



## Data Availability

The data underlying this study are available in the published article and its Supporting Information.

## Notes

Methods, instrumentation, synthetic procedures, schemes, figures, characterization data, including ^1^H NMR, ^13^C­{^1^H}-NMR, FTIR, and GPC, tables, and further comprehensive rationalizations on the complex reaction system (PDF)

## References

[1] Khairul
Anuar N. F. S., Huyop F., Ur-Rehman G., Abdullah F., Normi Y. M., Sabullah M. K., Abdul
Wahab R. (2022). An Overview into Polyethylene Terephthalate (PET) Hydrolases and
Efforts in Tailoring Enzymes for Improved Plastic Degradation. Int. J. Mol. Sci..

[2] Rashwan A. K., Younis H. A., Abdelshafy A. M., Osman A. I., Eletmany M. R., Hafouda M. A., Chen W. (2024). Plant starch
extraction, modification,
and green applications: a review. Environmental
Chemistry Letters.

[3] Di
Bartolo A., Infurna G., Dintcheva N. T. (2021). A Review
of Bioplastics and Their Adoption in the Circular Economy. Polymers.

[4] Zhao L., Zhou Y., Zhang J., Liang H., Chen X., Tan H. (2023). Natural Polymer-Based
Hydrogels: From Polymer to Biomedical Applications. Pharmaceutics.

[5] Oyekunle D. T., Nia M. H., Wilson L. D. (2024). Recent Progress on the Application
of Chitosan, Starch and Chitosan–Starch Composites for Meat
Preservation—A Mini Review. Journal of
Composites Science.

[6] Xu Y., Miladinov V., Hanna M. A. (2004). Synthesis and Characterization of
Starch Acetates with High Substitution. Cereal
Chem..

[7] García-Guzmán L., Cabrera-Barjas G., Soria-Hernández C. G., Castaño J., Guadarrama-Lezama A. Y., Rodríguez Llamazares S. (2022). Progress in
Starch-Based Materials for Food Packaging Applications. Polysaccharides.

[8] Nguyen M. T. P., Escribà-Gelonch M., Hessel V., Coad B. R. (2024). A Review
of the Current and Future Prospects for Producing Bioplastic Films
Made from Starch and Chitosan. ACS Sustainable
Chem. Eng..

[9] Fan Y., Picchioni F. (2020). Modification
of starch: A review on the application
of “green” solvents and controlled functionalization. Carbohydr. Polym..

[10] BeMiller J. N. (2018). Starch in Food;
Elsevier.

[11] Zarski A., Kapusniak K., Ptak S., Rudlicka M., Coseri S., Kapusniak J. (2024). Functionalization
Methods of Starch and Its Derivatives:
From Old Limitations to New Possibilities. Polymers.

[12] Xu H., Canisag H., Mu B., Yang Y. (2015). Robust and Flexible
Films from 100Starch Cross-Linked by Biobased Disaccharide Derivative. ACS Sustainable Chem. Eng..

[13] Moon S. H., Hwang H. J., Jeon H. R., Park S. J., Bae I. S., Yang Y. J. (2023). Photocrosslinkable
natural polymers in tissue engineering. Front.
Bioeng. Biotechnol..

[14] Dalle
Vacche S., Esposito L. H., Bugnotti D., Callone E., Orsini S. F., D’Arienzo M., Cipolla L., Petroni S., Vitale A., Bongiovanni R., Dirè S. (2025). Modification
of Epoxidized Soybean Oil for the Preparation of Amorphous, Nonretrogradable,
and Hydrophobic Starch Films. Polysaccharides.

[15] Orsini S. F., Cipolla L., Petroni S., Dirè S., Ceccato R., Callone E., Bongiovanni R., Dalle Vacche S., Di Credico B., Mostoni S., Nisticò R., Raimondo L., Scotti R., D’Arienzo M. (2022). Synthesis
and Characterization of Alkoxysilane-Bearing Photoreversible Cinnamic
Side Groups: A Promising Building-Block for the Design of Multifunctional
Silica Nanoparticles. Langmuir.

[16] Zhang R., Chu F., Hu Y., Hu H., Hu Y., Liu H., Huo C., Wang H. (2020). Preparation
of Photo-Crosslinking Starch Colloidal
Particles. Starch - Stärke.

[17] Petroni S., Orsini S. F., Bugnotti D., Callone E., Dirè S., Zoia L., Bongiovanni R., Dalle Vacche S., Vitale A., Raimondo L., Sassella A., Mariani P., D’Arienzo M., Cipolla L. (2025). Photocrosslinkable
starch cinnamyl
ethers as bioinspired bio-based polymers. J.
Mater. Chem. B.

[18] Huijbrechts A. M., Vermonden T., Bogaert P., Franssen M. C., Visser G. M., Boeriu C. G., Sudhölter E. J. (2009). Optimization of the synthesis of
1-allyloxy-2-hydroxy-propyl-starch through statistical experimental
design. Carbohydr. Polym..

[19] Leardi R. (2009). Experimental
design in chemistry: A tutorial. Anal. Chim.
Acta.

[20] Ortiz-Fernández A., Carrillo-Sánchez F., May-Hernández L., Estrada-León R., Carrillo-Escalante H., Hernández-Sánchez F., Valadez-Gonzalez A. (2017). Design of experiments for optimization a biodegrable
adhesive based on ramon starch (Brosimum alicastrum Sw.). Int. J. Adhes. Adhes..

[21] Still W. C., Kahn M., Mitra A. (1978). Rapid chromatographic
technique for
preparative separations with moderate resolution. J. Org. Chem..

[22] Falsafi S. R., Maghsoudlou Y., Aalami M., Jafari S. M., Raeisi M. (2019). Physicochemical
and morphological properties of resistant starch type 4 prepared under
ultrasound and conventional conditions and their in-vitro and in-vivo
digestibilities. Ultrasonics Sonochemistry.

[23] Ai Y., Jane J. (2015). Gelatinization and
rheological properties of starch. Starch - Stärke.

[24] Morris V. (1990). Starch gelation
and retrogradation. Trends in Food Science &
Technology.

[25] JMP Pro, Version 18.0.2. 2024.

[26] Tankam P. F., Müller R., Mischnick P., Hopf H. (2007). Alkynyl polysaccharides:
synthesis of propargyl potato starch followed by subsequent derivatizations. Carbohydr. Res..

[27] Zhao W., Kloczkowski A., Mark J. E., Erman B. (1998). Novel High-Performance
Materials from Starch. 1. Factors Influencing the Lyotropic Liquid
Crystallinity of Some Starch Ethers. Chem. Mater..

[28] Chi C., He Y., Jiao W., Wang H., Tan X. (2022). Hierarchical structural
transformation of corn starch in NaOH solution at room temperature. Industrial Crops and Products.

[29] Conant J. B., Tuttle N. (1921). MESITYL OXIDE. Org. Synth..

[30] Cataldo F. (1996). Synthesis
of ketonic resins from self-polymerization of acetone, 2. Action of
bases on acetone and the synthesis of halogenated and diels-alder
adducts. *Die*. Angew. Makromol.
Chem..

[31] Ruther T., Müller M.-A., Bonrath W., Eisenacher M., Ruther T., Müller M.-A., Bonrath W., Eisenacher M. (2023). The Production
of Isophorone. Encyclopedia.

[32] Zhang Z.-W., Li H.-B., Li J., Wang C.-C., Feng J., Yang Y.-H., Liu S. (2020). Synthesis of Epoxides
from Alkyl
Bromides and Alcohols with in Situ Generation of Dimethyl Sulfonium
Ylide in DMSO Oxidations. J. Org. Chem..

[33] Qin Y., Zhang H., Dai Y., Hou H., Dong H. (2019). Effect of
Alkali Treatment on Structure and Properties of High Amylose Corn
Starch Film. Materials.

[34] Yoo W., Yoo D., Hong E., Jung E., Go Y., Singh S. B., Khang G., Lee D. (2018). Acid-activatable oxidative stress-inducing
polysaccharide nanoparticles for anticancer therapy. J. Controlled Release.

