# Supplementary material for: Statistical Investigation and Optimization of Starch Cinnamylation: A Design of Experiment Approach
Source: ACS Omega. 2026 Apr 10;11(15):23211–26. doi: 10.1021/acsomega.5c13678 (PMC13103797; doi:10.1021/acsomega.5c13678)
Supplement: Supplementary file 1 [file ao5c13678_si_001.pdf]

Supplementary Material

Statistical Investigation and Optimization of Starch Cinnamylation: A Design of Experiment Approach

Luca Leuzzi[a], Laura Cipolla[a]*

[a] Department of Biotechnology and Biosciences, University of Milano-Bicocca, Piazza della Scienza 2, 20126 Milano, Italy
Corresponding authour: Prof. Laura Cipolla, e-mail: laura.cipolla@unimib.it

---

Summary

S1 Experimental, Materials and Methods **3**
S1.1 Base preparation . . . 3
S1.1.1 Sodium Dimsyl DMSO solution (2 M) . . . 3
S1.1.2 NaOH dispersion in DMSO (5 M) . . . 3
S1.1.3 Sodium Alkoxides - methoxide, ethoxide, and iso-propoxide . . . 3
S1.2 Spectroscopic characterization - NMR and FTIR . . . 3
S1.2.1 Degree of substitution (DS) calculation by NMR . . . 3
S1.2.2 Degree of branching (DB) calculation by NMR . . . 4
S1.3 Potato starch benzoylation for gel permeation chromatography . . . 4
S1.4 DoE and statistical analysis . . . 5
S1.4.1 Screening Phase: Full Factorial Design approach . . . 5
S1.4.2 Optimization Phase: Central Composite Design approach . . . 6

S2 Supporting Schemes **7**

S3 Supporting Figures **8**

S4 Starch GPC characterization **10**

S5 NMR spectra **11**
S5.1 ^{1}H-NMR of pristine potato starch . . . 11
S5.2 ^{13}C-NMR of pristine potato starch . . . 12
S5.3 ^{1}H-NMR of cinnamyl alcohol (CINN-OH) . . . 12
S5.4 ^{1}H-NMR of dicinnamyl ether (CINN-O-CINN) . . . 13
S5.5 ^{1}H-NMR of base treated starch . . . 13
S5.6 ^{1}H-NMR of cinnamylated starches at different DS . . . 14
S5.6.1 ^{1}H-NMR of cinnamylated starch with DS of 0.4910 . . . 14
S5.6.2 ^{1}H-NMR of cinnamylated starch with DS of 0.9716 . . . 15
S5.6.3 ^{1}H-NMR of cinnamylated starch with DS of 1.3318 . . . 15
S5.6.4 ^{1}H-NMR of cinnamylated starch with DS of 1.6218 . . . 16
S5.6.5 ^{1}H-NMR of cinnamylated starch with DS of 1.7776 . . . 16
S5.6.6 ^{1}H-NMR of cinnamylated starch with DS of 1.8164 . . . 17
S5.6.7 ^{1}H-NMR of cinnamylated starch with DS of 1.9790 . . . 17
S5.6.8 ^{1}H-NMR of cinnamylated starch with DS of 2.0682 . . . 18
S5.6.9 ^{1}H-NMR of cinnamylated at different DS . . . 18

S6 NaOH side-reactions **19**

S7 Supporting Tables 20
S7.1 Base variation 20

S8 Full Factorial Design - Screening Phase 20
S8.1 SP1-DoE 20
S8.2 SP2-DoE 20
S8.3 SP3-DoE 21

S9 Central Composite Design - Optimization Phase 22
S9.1 Estimated Coefficients Effects “Solid a” DS 22
S9.2 Estimated Coefficients Effects “Solid b” DS 22
S9.3 Estimated Coefficients Effects “Solid a” Weight 23
S9.4 Estimated Coefficients Effects “Solid b” Weight 23

S10 Model validation 24

S1 Experimental, Materials and Methods

S1.1 Base preparation

S1.1.1 Sodium Dimsyl DMSO solution (2 M)

A double-necked round-bottom flask (25 mL) is equipped with a magnetic stirrer, 800 mg of NaH (60% dispersion in mineral oil, 20 mmol), a reversible rubber cap, and a three-way tap connected with one end to a water aspirator, and the other to a balloon filled with argon gas. The system is then cycled with inert gas. Afterwards, 10 mL of petroleum ether are added to NaH and stirred; subsequently, it is left to decant, the solvent removed, and NaH dried. This procedure was repeated twice. Afterwards, 10 mL of dry DMSO are gently added and the mixture is carefully heated up to 75 °C for 1 hour. Finally, the olive-grey obtained solution is cooled down to room temperature and used immediately.

S1.1.2 NaOH dispersion in DMSO (5 M)

2 grams of NaOH (MW = 39.997 g·mol^{-1}, 50 mmol) are carefully dissolved in 15 mL of dry MeOH in a water bath initially at room temperature, which was than slightly heated to promote the complete dissolution; then, 10 mL of dry DMSO are added and mixed. Finally, the alcohol is removed at reduced pressure, leading to a viscous and cloudy mixture which was used without further purification.

S1.1.3 Sodium Alkoxides - methoxide, ethoxide, and iso-propoxide

Typically, an excess of the title alcohol is poured into a round-bottom flask equipped with a water bath, a magnetic stirrer, and an Allihn condenser. Then, metallic sodium is carefully added in tiny fragments. The mixture is left reacting until complete dissolution of sodium. Then, the alcohol excess is removed at reduced pressure, leaving a white to beige precipitate, which is stored under inert gas in a moisture-free container. The alkoxides are then used without any further processing.

S1.2 Spectroscopic characterization - NMR and FTIR

The NMRs were recorded with a Bruker Avance 400 WB spectrometer operating at the proton frequency of 400.13 MHz for ^{1}H-NMR and at the carbon frequency of 100.61 MHz for the ^{13}C-NMR with complete proton decoupling. Proton and carbon chemical shifts are reported in ppm (δ) using solvent residual peaks as internal reference ($CDCl_3$ δ = 7.26 / 77.16 ppm; $(CD_3)_2SO$ δ = 2.50 / 39.52 ppm). The following abbreviations are used to describe spin multiplicity: s = singlet, d = doublet, t = triplet, q = quartet, m = multiplet, brs = broad signal, dd = doublet-doublet, td = triplet-doublet, ddd = doublet-doublet-doublet. Coupling constant (J) values are reported in Hz. The spectra were elaborated with the TopSpin Bruker software.
The starch-derived analyte is generally prepared by dissolving ≈ 20 mg of sample in 750 μL of DMSO-d^6 at 80-90 °C under stirring until dissolution (for the more substituted products, high temperatures weren't required). To this, at room temperature 50 μL of TFA-d were added to promote OH proton to deuterium exchange to quench hydroxy group signals in order to easily determine the DS.
Fourier transform infrared spectra were recorded with a Jasco Inc FT/IR - 4100 analyzer with the additional external module ATR PRO One, within the wavenumber full range of 399.193 to 7800.65 cm^{-1} by setting the instrument to the automatic scan mode (recording a minimum number of 50 scans) at a resolution of 4 cm^{-1}. Background spectra were firstly collected, at room temperature, before each measurement and then automatically subtracted from each sample spectra by the software.

S1.2.1 Degree of substitution (DS) calculation by NMR

The DS was calculated by exploiting the ratio between the integration area of the cinnamyl moiety aromatic protons and (A_{Ph}/5) and the integration areas of the anomeric protons ($A_{\alpha(1,4)}$+$A_{\alpha(1,6)}$) in the NMR following the equation (S1):

$$DS = \frac{A_{Ph}}{5 \cdot (A_{\alpha(1,4)} + A_{\alpha(1,6)})} \tag{S1}$$

The DS defines the average number of substituents per anhydroglucopyranose unit (starch-AGU) for the hydroxy group in positions 2, 3, and 6; therefore, it oscillates between 0.0 and its maximum theoretical value of 3.0, which cannot be obtained due to the amylopectin branches. Significant NMR spectra are reported in Figures S11-S18.

S1.2.2 Degree of branching (DB) calculation by NMR

The DB % can be determined by the ratio between the integration area of the anomeric protons involved in the α-1,6 linkages, and the integration area sum of the anomeric protons, following the equation (S2):

$$DB(\%) = \frac{A_{\alpha(1,6)}}{A_{\alpha(1,4)} + A_{\alpha(1,6)}} \cdot 100 \tag{S2}$$

S1.3 Potato starch benzoylation for gel permeation chromatography

Perbenzoylated starch was prepared to perform GPC analysis, it was prepared by slightly adapting the experimental procedure of Zoia *et al.* [1].

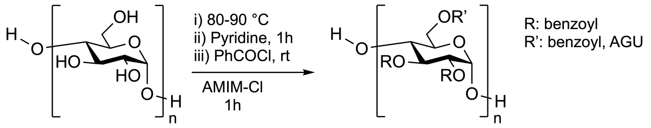

Scheme S1: Starch benzoylation reaction scheme.

100 mg (0.617 mmol starch-AGU) of dried potato starch are dissolved in 1.0 g of AMIM-Cl in a round-bottom flask (25 mL) at 80-90 °C, previously heated in a static oven at 105 °C for 48 h, by severe magnetic stirring until complete dissolution. Then, the mixture is cooled to room temperature. Subsequently 745 μL of pyridine (9.25 mmol) are added into the main batch, and the mixture is vortexed at room temperature to afford a homogeneous solution. Afterwards, 610 μL of PhCOCl (5.24 mmol) are carefully added dropwise under magnetic stirring. The crude product is precipitated by adding 20 mL of a 7:3 EtOH:H_2O mixture. The solids are recovered by centrifugation (6000 rpm, 15 °C, 5 min), after removing the supernatant. In order to fully remove the ionic liquid, pyridine salts and excess reagents, the precipitate is washed twice with a 7:3 fresh mixture of EtOH:H_2O (20 mL) followed by centrifugation (6000 rpm, 15 °C, 5 min). The precipitate is then finally recovered by solvent removal, and air drying in the fume hood.

Benzoylated potato starch characterization:

^{1}H-NMR (DMSO-d^6 + TFA-d, 400 MHz) δ[ppm]: 8.15 - 7.00 (brs, 5H; =C**H**), 5.11 (brs, 1H, -C_1**H**); 3.76 - 3.50 (brs, 3H, -$C_{3,5,6}$**H**); 3.42 - 3.22 (brs, 2 H, -$C_{2,4}$**H**); spectrum (not shown) coherent with the information reported in the literature [2].

FT-IR (Figure S1): absence of free -OH bands between 3000 and 3700 cm^{-1}; 1717 cm^{-1} (C=O vibration of the benzylic ester group); 1450 cm^{-1} (aromatic C=C stretching); 1264 cm^{-1} (C-O aromatic ester stretching); 706 cm^{-1} (aromatic C-H out ofplane stretching).

Both characterization techniques confirmed the correct functionalization of starch.

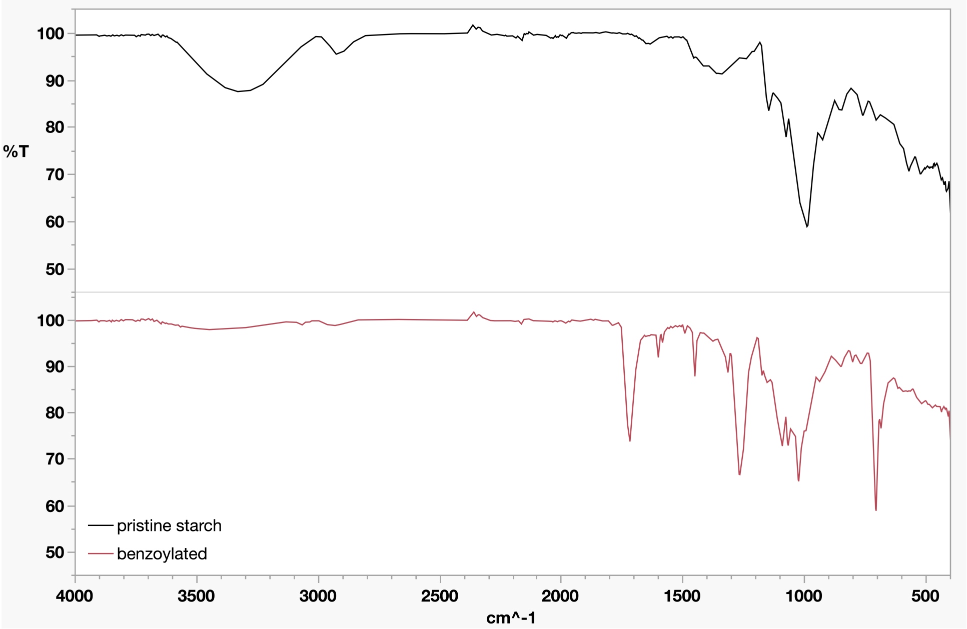

Figure S1: FT-IR (ATR) of pristine starch compared with benzoylated starch.

The prepared benzoylated starch was then analyzed by GPC to determine the respective Mw (g/mol) as described in Section S4.

S1.4 DoE and statistical analysis

The experimental designs and statistical analysis of the data were carried out using the software JMP Pro 18 (JMP Pro, Version 18.0.2, 2024), according to the significance level established to obtain the mathematical model. In both screening and optimization processes, the experiments were conducted in a randomized order as dictated by the experimental design software. The statistical validity was evaluated by examining its coefficient of determination (R^2), which quantifies the proportion of variability in the observed data that is explained by the model. The R^2 values may range from 0 to 1, with values approaching to 1 indicating greater model reliability. An improvement in R^2 typically occurs upon the removal of non-significant factors; however, in this study, no notable enhancements in R^2 were observed following the exclusion of any factors. Consequently, all parameters were retained and included in the models.

S1.4.1 Screening Phase: Full Factorial Design approach

A Full Factorial Design (FFD) in screening mode was carried out to investigate the role of the factors and their interactions on two levels, respectively "-1" and "$+1$". The equations that the model built to describe the systems can be generally written as follows:

$$y = \beta_0 + \beta_1 X_1 + \beta_2 X_2 + \beta_{12} X_{12} \tag{S3}$$

$$y = \beta_0 + \beta_1 X_1 + \beta_2 X_2 + \beta_3 X_3 + \beta_{12} X_{12} + \beta_{13} X_{13} + \beta_{23} X_{23} + \beta_{123} X_{123} \tag{S4}$$

Eq. S3 and Eq. S4 differ from the number of factors in the FFD, respectively. The "y" represents the response variable, "β_0" is the intercept or the constant term in the model, "X_n" indicates the different factors with their relative coefficients "β_n", and "X_{nm}" represents the interaction factor between factor "X_n" and "X_m".

S1.4.2 Optimization Phase: Central Composite Design approach

A Central Composite Design with 5 center points was performed to further investigate two factors, namely the amounts of NaOH and cinnamyl chloride at five levels (±1, 0, and $\pm\alpha$), considering the DS and final weight as responses. To fit the equation of the model, which can be written as follows:

$$y = \beta_0 + \beta_1 X_1 + \beta_2 X_2 + \beta_{12} X_{12} + \beta_1^2 X_1^2 + \beta_2^2 X_2^2 + \varepsilon \quad \text{(S5)}$$

standard least square (OLS) regression was used. The "y" represents the response, "β_x" are the coefficients of single factors "X_n", of interaction ones "X_{nm}", and of the quadratic terms "X_n^2". Finally, the term "ε" represents the associated error to the model.
A CCD-Uniform precision model was used, as suggested by the software, which indicated a total of 13 experiments. The "$\pm\alpha$" points were calculated with the square root of the number of evaluated factors ($\sqrt{k} = 1.4142\ldots$, where k = 2).

S2 Supporting Schemes

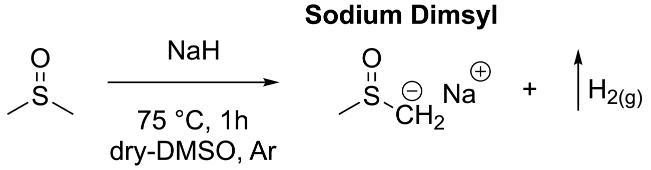

Scheme S2: Sodium dimsyl anion preparation.

S3 Supporting Figures

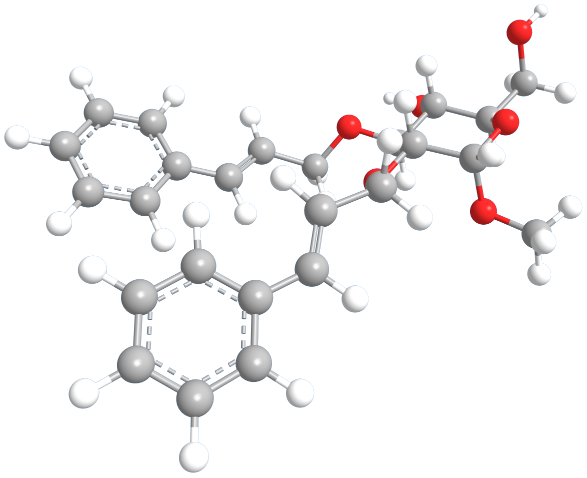

Figure S2: Aromatic rings steric hindrance between vicinal positions 2 and 3 on α-methyl glucoside as reference model (ChemBio3D Version 14.0.0.117, 2014, CambridgeSoft Corp., PerkinElmer).

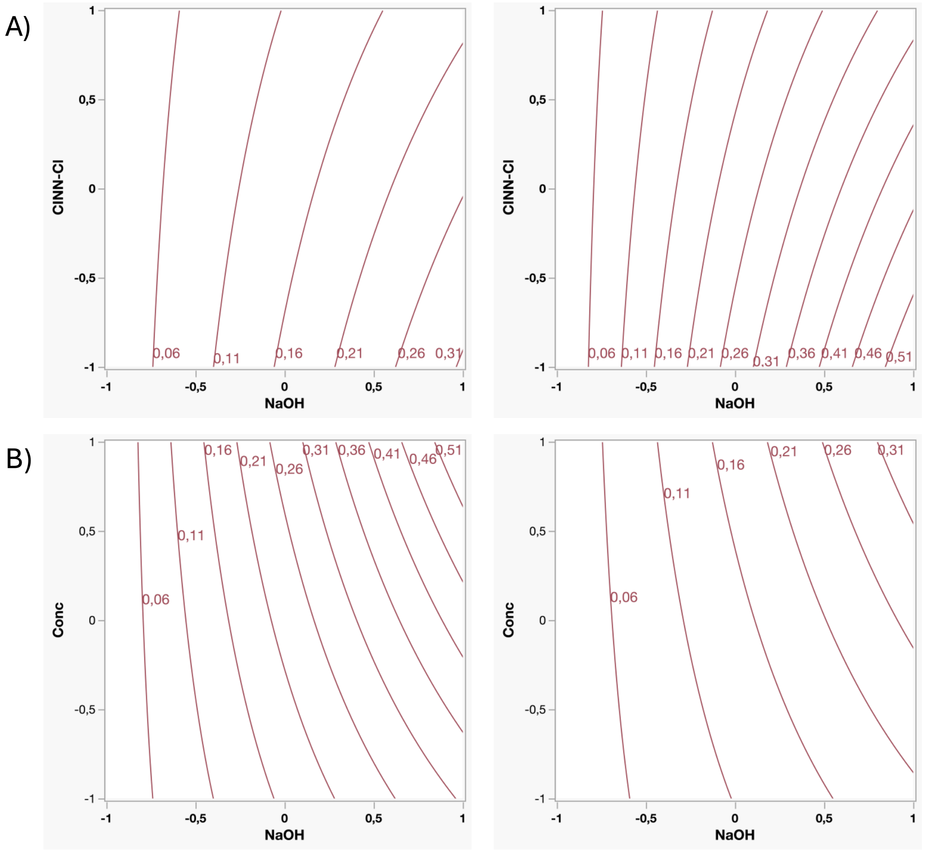

Figure S3: FFD Contour Plots of [NaOH*CINN-Cl] (A) and [NaOH*Conc] (B) interaction factors, fixed at Conc "-1" and on "+1" respectively on left and right.

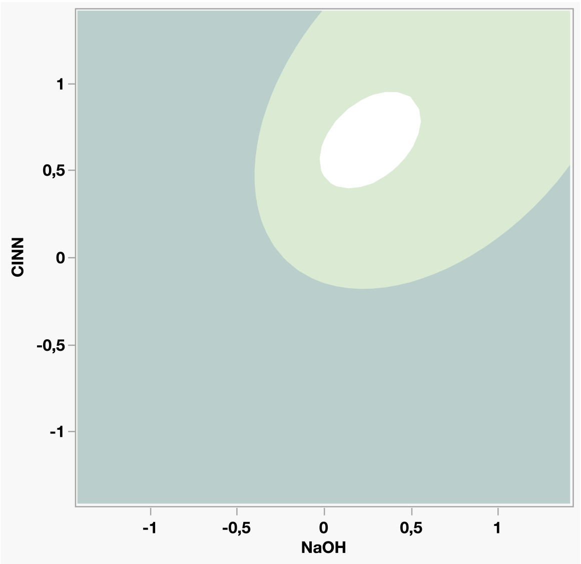

Figure S4: Overlapped responses contour plots identifying the experimental space (white area) to achieve the desired DS, resulting by limiting the formation of “solid b” > 500 mg, and “solid a”< 120 mg.

S4 Starch GPC characterization

To prepare the GPC sample, 1 mg of perbenzoylated starch is dissolved in THF and filtered with a GHP 0.45 mm Acrodis syringe filter for the GPC analysis [3]. The analyses are performed with a HP1100 series liquid chromatography connected to a HP 1040 UV photodetector at a wavelength of 240 nm. The injector has a Rheodyne loop valve with a loop capacity of 20 mL. The GPC-column system was composed as follows (according to the solvent flow direction): Agilent PL gel 5 mm (500 Å), Agilent PL gel 5 mm (1000 Å) and Agilent PL gel 5 mm (10000 Å). THF at a flow rate of 1 $mL \cdot min^{-1}$ was flushed. PL Polymer Standards of Polystyrene from Polymer Laboratories are used for calibration. The evaluation of the number-average molecular weight (Mn) and the weight-average molecular weight (Mw) of the samples is performed. Moreover, the ratio D = Mw/Mn, defined as the dispersity index is also calculated. The reported Mn and Mw values are the average of three analyses (standard error Mw: 500 g/mol; Mn: 100 g/mol).
The subsequent GPC analysis (Figure S5) allowed the determination of the number-average molecular weight (Mn), the weight-average molecular weight (Mw), and the peak molecular weight (Mp), which is defined as the molecular weight at the maximum absorbance, along with the dispersity index (D).

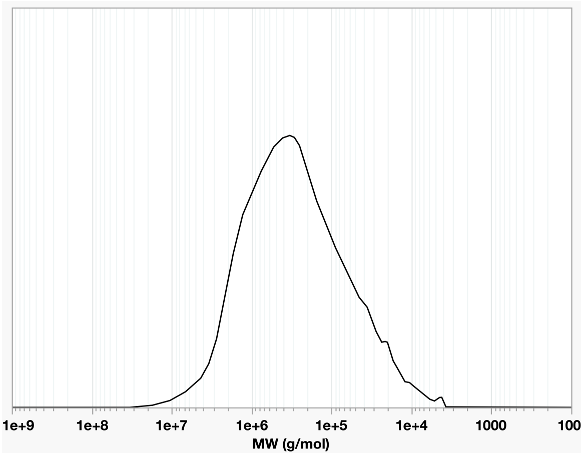

Figure S5: GPC chromatogram of benzoylated potato starch.

The GPC profile displayed a polymodal distribution, affording different values: Mw = $2.37 \cdot 10^6$ Da, Mn = $4.74 \cdot 10^5$ Da, Mp = $3.29 \cdot 10^5$ Da, D = 4.99.
The peak is constituted by different overlapped signals, that are related to the starch components: amylose and amylopectin, where the latter is typically more heavier than the linear fraction.

S5 NMR spectra

S5.1 ^{1}H-NMR of pristine potato starch

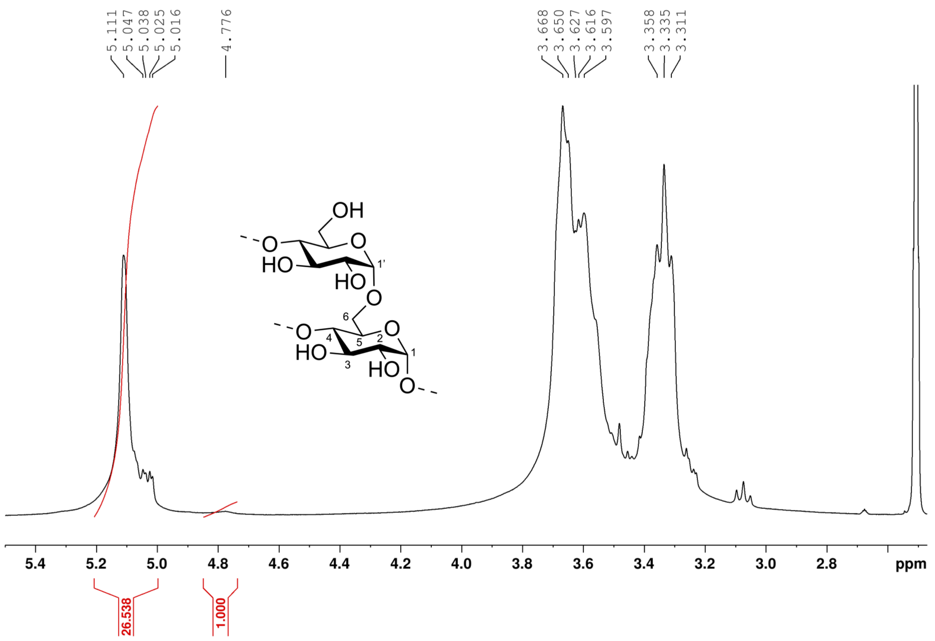

Figure S6: ^{1}H-NMR of pristine potato starch in DMSO-d^6 + TFA-d

^{1}H-NMR (DMSO-d^6 + TFA-d, 400 MHz) δ [ppm]: 5.11 - 5.02 (brs, 1H - H_1); 4.78 (s, 1H - $H_{1'}$); 3.67 - 3.59 (brs, 3H - $H_{3,5,6}$); 3.36 - 3.31 (brs, 2H - $H_{2,4}$).

The DB% was calculated as follows:

$$DB(\%) = \frac{A_{(\alpha 1-6)}}{A_{(\alpha 1-6)} + A_{(\alpha 1-4)}} \cdot 100 = 3.631\% \tag{S6}$$

S5.2 ^{13}C-NMR of pristine potato starch

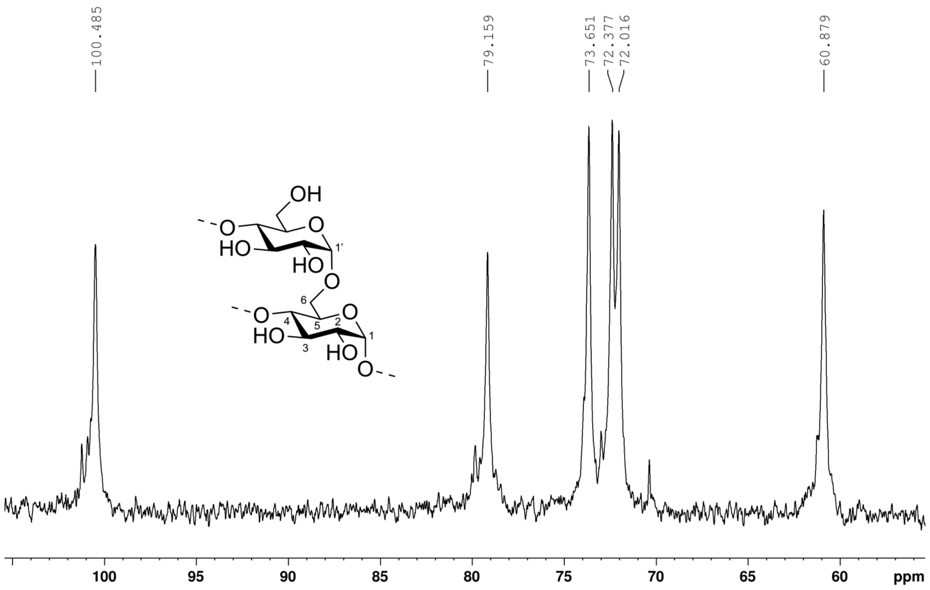

Figure S7: ^{13}C{^{1}H}-NMR of pristine potato starch in DMSO-d^6

^{13}C{^{1}H}-NMR (DMSO-d^6, 100 MHz) δ [ppm]: 100.49 (1C - C_1); 79.16 (1C - C_4); 73.65 (1C - C_5); 72.38 (1C - C_3); 72.02 (1C - C_2); 60.88 (1C - C_6).

S5.3 ^{1}H-NMR of cinnamyl alcohol (CINN-OH)

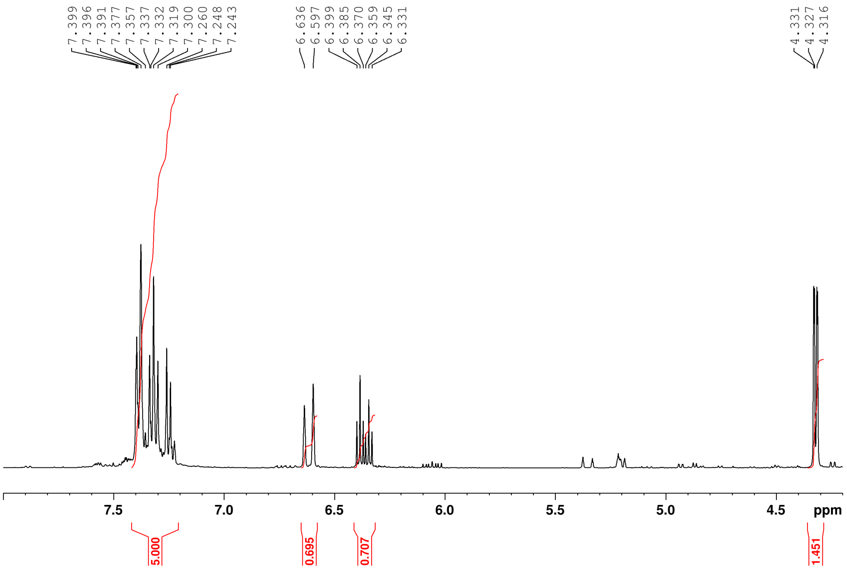

Figure S8: ^{1}H-NMR of cinnamyl alcohol in $CDCl_3$

^{1}H-NMR ($CDCl_3$, 400 MHz) δ [ppm]: 7.40-7.38 (m, 2 H), 7.36-7.30 (m, 2 H), 7.26-7.24 (m, 1 H), 6.62 (d, J = 15.9 Hz, 1 H), 6.36 (dt, J = 15.9, 5.7 Hz, 1 H), 4.32 (dd, J = 5.7, 1.4 Hz, 2 H).

S5.4 ^{1}H-NMR of dicinnamyl ether (CINN-O-CINN)

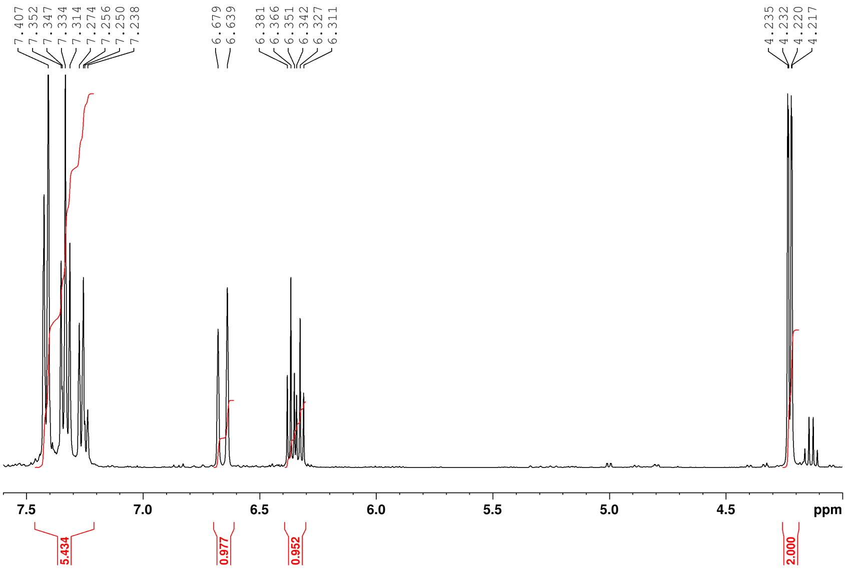

Figure S9: ^{1}H-NMR of dicinnamyl ether in $CDCl_3$

^{1}H-NMR ($CDCl_3$, 400 MHz) δ [ppm]: 7.43-7.41 (m, 2 H), 7.35-7.33 (m, 2 H), 7.27-7.24 (m, 1 H), 6.66 (d, J = 15.9 Hz, 1 H), 6.35 (dt, J = 15.9, 6.1 Hz, 1 H), 4.23 (dd, J = 6.1, 2.3 Hz, 2 H).

S5.5 ^{1}H-NMR of base treated starch

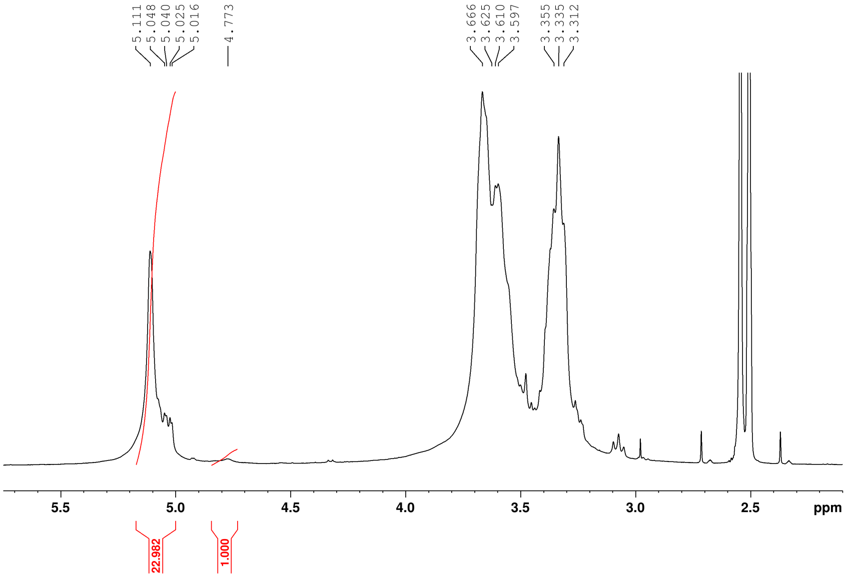

Figure S10: ^{1}H-NMR of base treated potato starch in DMSO-d^6 + TFA-d

^{1}H-NMR (DMSO-d^6 + TFA-d, 400 MHz) δ [ppm]: 5.11 - 5.02 (brs, 1H); 4.77 (s, 1H); 3.67 - 3.60 (brs, 3H); 3.36 - 3.31 (brs, 2 H).

The DB% was calculated as follows:

$$DB(\%) = \frac{A_{(\alpha 1-6)}}{A_{(\alpha 1-6)} + A_{(\alpha 1-4)}} \cdot 100 = 4.170\% \qquad \text{(S7)}$$

S5.6 ¹H-NMR of cinnamylated starches at different DS

S5.6.1 ¹H-NMR of cinnamylated starch with DS of 0.4910

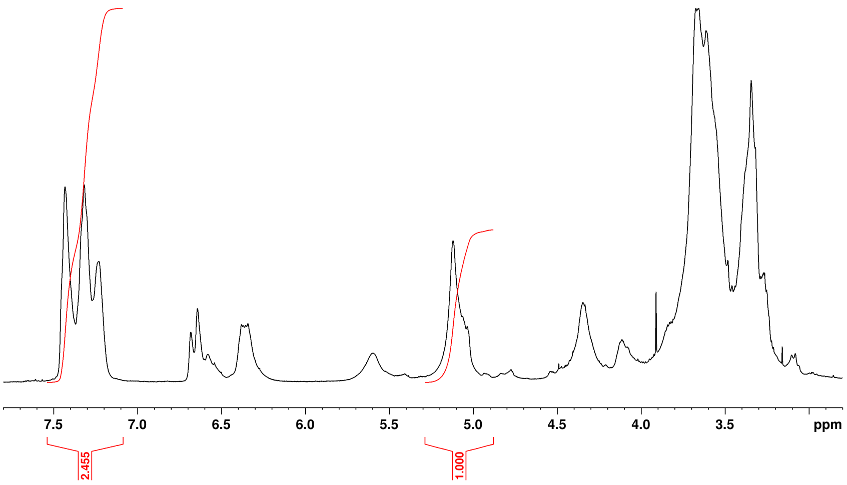

Figure S11: ^{1}H-NMR of cinnamylated starch in DMSO-d^6 + TFA-d.

S5.6.2 ^{1}H-NMR of cinnamylated starch with DS of 0.9716

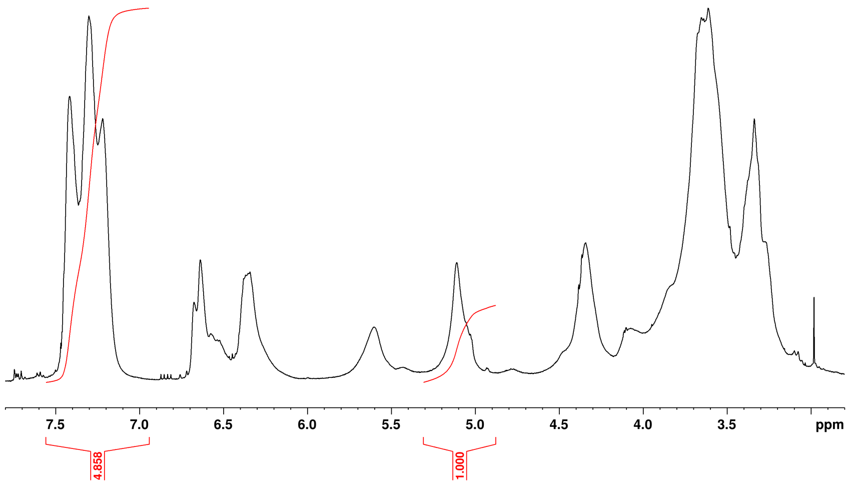

Figure S12: ^{1}H-NMR of cinnamylated starch in DMSO-d^{6} + TFA-d.

S5.6.3 ^{1}H-NMR of cinnamylated starch with DS of 1.3318

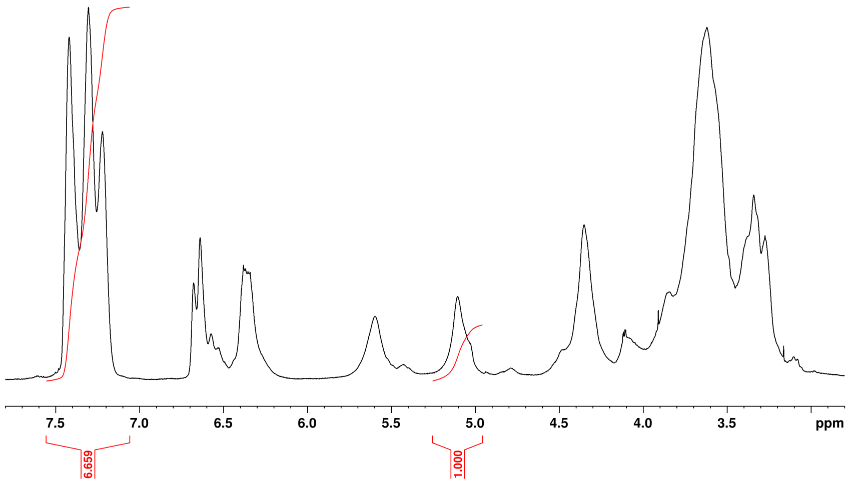

Figure S13: ^{1}H-NMR of cinnamylated starch in DMSO-d^{6} + TFA-d.

S5.6.4 ^{1}H-NMR of cinnamylated starch with DS of 1.6218

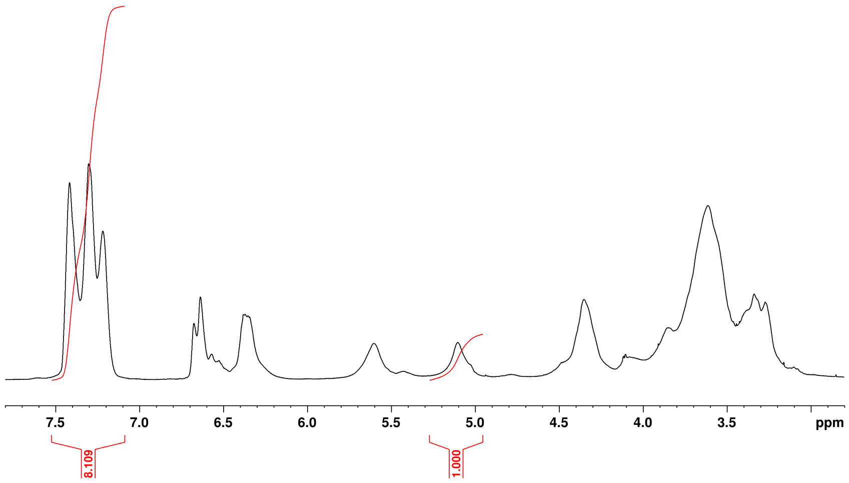

Figure S14: ^{1}H-NMR of cinnamylated starch in DMSO-d^6 + TFA-d.

S5.6.5 ^{1}H-NMR of cinnamylated starch with DS of 1.7776

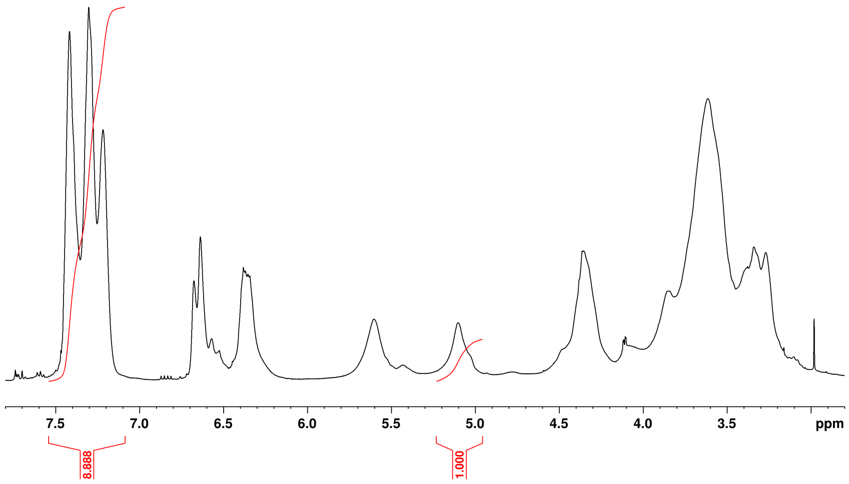

Figure S15: ^{1}H-NMR of cinnamylated starch in DMSO-d^6 + TFA-d.

S5.6.6 ^{1}H-NMR of cinnamylated starch with DS of 1.8164

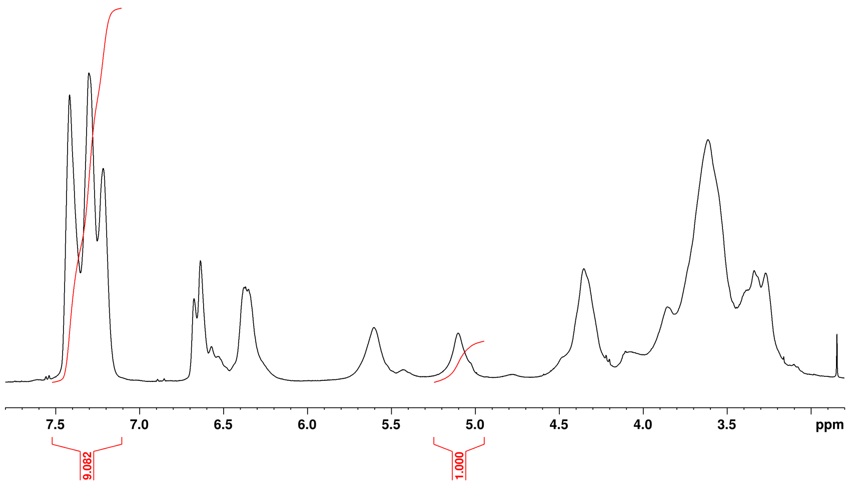

Figure S16: ^{1}H-NMR of cinnamylated starch in DMSO-d^{6} + TFA-d.

S5.6.7 ^{1}H-NMR of cinnamylated starch with DS of 1.9790

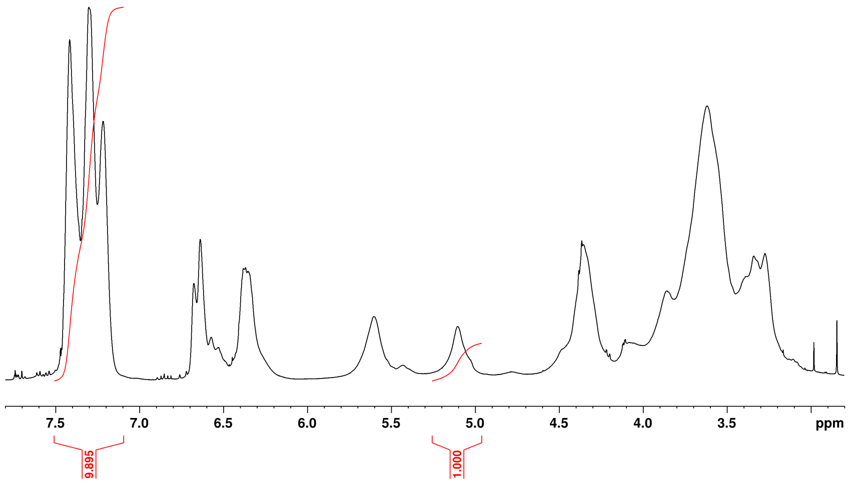

Figure S17: ^{1}H-NMR of cinnamylated starch in DMSO-d^{6} + TFA-d.

S5.6.8 ^{1}H-NMR of cinnamylated starch with DS of 2.0682

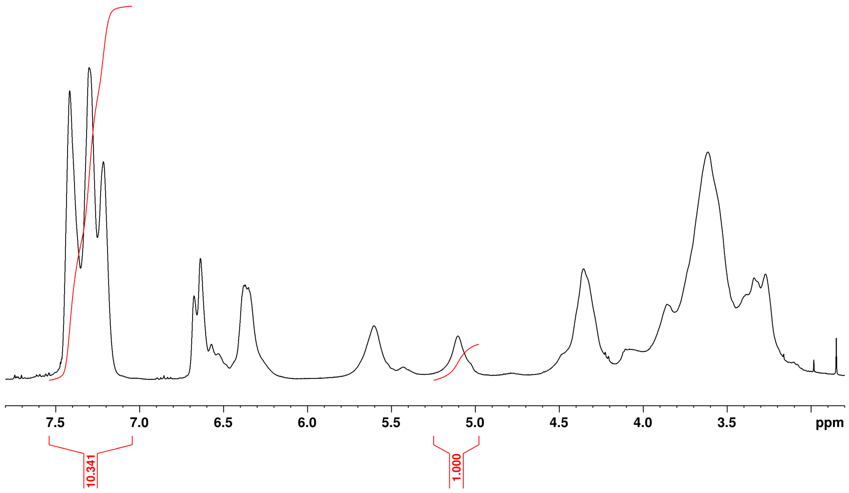

Figure S18: ^{1}H-NMR of cinnamylated starch in DMSO-d^6 + TFA-d.

S5.6.9 ^{1}H-NMR of cinnamylated at different DS

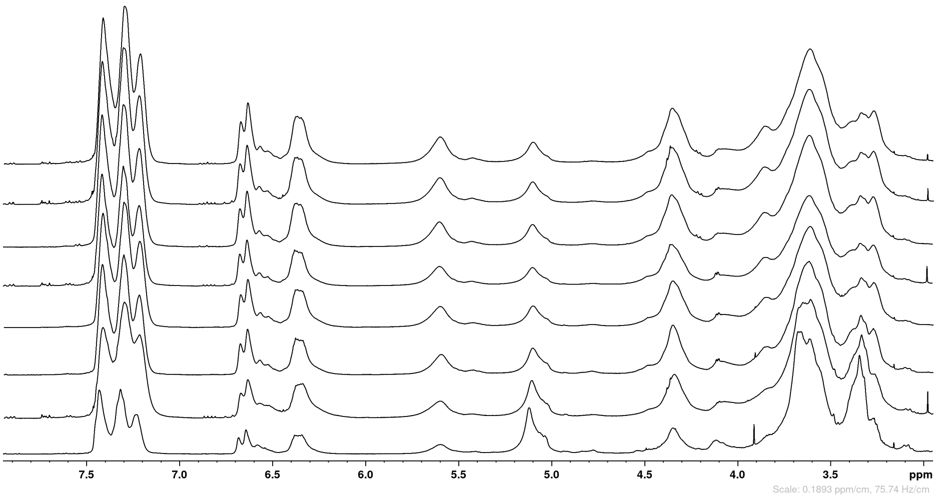

Figure S19: ^{1}H-NMR comparison of cinnamylated starches. From bottom to the top: 0.4910, 0.9716, 1.3318, 1.6218, 1.7776, 1.8164, 1.9790, 2.0682, respectively.

S6 NaOH side-reactions

To evaluate whether NaOH might promote side-reactions on starch beyond merely deprotonating its hydroxy groups, an experimental procedure aligned with the optimization protocol was performed (1.0 equivalents of starch-AGU - 60 g/L, 3.0 equivalents of NaOH dispersed in DMSO). The reaction was terminated immediately after the base treatment (1 h) by neutralizing the mixture with a 1.0 M aqueous HCl solution. The resulting solid was subsequently isolated via precipitation in cold acetone, as previously detailed in the Experimental Section of the manuscript. Neutralization of the base required 3.7 mmol (3.7 mL) of acid to attain an indicative pH of 7. However, the experimental amount of base corresponds to 9.25 mmol. This observation suggests a significant depletion of base (5.55 mmol) from the reaction system that might be ascribed to side reactions, such as polysaccharide degradation [4]. As evidenced by the ^{1}H-NMR analysis (Figure S10), the respective degree of branching (DB%) following base treatment resulted higher than pristine starch. This increase may be directly associated with the cleavage of α-1,4-glycosidic bonds. Nonetheless, such a substantial depletion of base would undoubtedly adversely affect the reaction system, notably limiting the maximum achievable degree of substitution (DS).
Regrettably, this changes were not easily detectable by the classical spectroscopic techniques, as IR and NMR, where the base-treated sample resulted to be extremely similar, if not perfectly identical, to the pristine polysaccharide. However, to further confirming the occurring of starch structure disruption process, it was though to analyze by GPC the treated starch. Thus, the base-treated starch was benzoylated, as described in Section S1.3, and than analyzed with the GPC as discussed in Section S4; the chromatogram is reported in Figure S20.

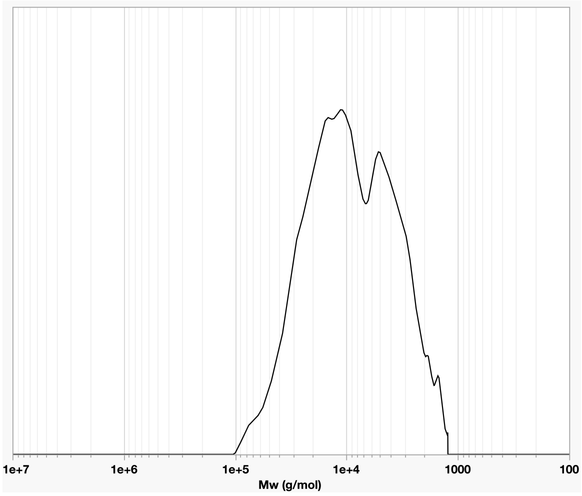

Figure S20: GPC chromatogram of benzoylated base-treated starch.

Compared to the untreated starch, the polysaccharide molecular weight was significantly decreased; as a fact, it resulted Mw = $2.38 \cdot 10^4$ Da (and an Mn = $1.29 \cdot 10^4$ Da), which can be approximately converted into a breakage of 4/5 random glycosidic bonds around the polysaccharide backbone. This result, alongside the other evidence on the favor of our hypotheses, confirmed the partial degradation of starch molecular structure, leading to a situation where part of the base is irreversibly consumed, and the substrate is partially converted into something slightly different which also might be able to participate in other processes (Section 4).

S7 Supporting Tables

S7.1 Base variation

Table S1: Different base results.

Entry	Base	Experimental DS[a]	ΔDS[b] (%)
control	NaOH	0.55	/
1	NaOMe	0.32	−23.4
2	NaOEt	0.59	+3.5
3	NaO*i*-Pr	0.67	+11.2
4	NaDMSO	0.77	+21.5
5	$NaOH_{(DMSO)}$	0.98	+42.7

**react. cond.: base (3.0 equiv.), CINN-Cl (1.0 equiv.), 60 g/L*

[a]*determined by NMR*

[b]*percentage increment of achieved DS.*

S8 Full Factorial Design - Screening Phase

S8.1 SP1-DoE

Table S2: Experimental and coded values

Variables	−1	+1
NaOH (equiv.)	1.0	3.0
CINN-Cl (equiv.)	1.0	3.0
Conc ($g{\cdot}L^{-1}$)	20	60

Table S3: FFD design matrix.

Entry	NaOH (equiv.)	CINN-Cl (equiv.)	Conc ($g{\cdot}L^{-1}$)
1	3.0	1.0	60
2	1.0	3.0	60
3	3.0	3.0	60
4	3.0	3.0	20
5	1.0	1.0	20
6	1.0	1.0	60
7	1.0	3.0	20
8	3.0	1.0	20

S8.2 SP2-DoE

Table S4: FFD factors combination.

Entry	NaOH (equiv.)	CINN-Cl (equiv.)	CINN-X (type)
1	1.0	1.0	Chloride
2	3.0	1.0	Chloride
3	1.0	3.0	Bromide
4	1.0	1.0	Bromide
5	1.0	3.0	Chloride
6	3.0	1.0	Bromide
7	3.0	3.0	Chloride
8	3.0	3.0	Bromide

Table S5: Experimental and coded values

Variables	−1	+1	Chloride	Bromide
NaOH (equiv.)	1.0	3.0	/	/
CINN-Cl (equiv.)	1.0	3.0	/	/
CINN-X	/	/	Cl	Br

S8.3 SP3-DoE

Table S6: FFD factors combination.

Entry	Temp (°C)	Addition
1	rt	portion-wise
2	rt	one-shot
3	90	one-shot
4	90	portion-wise

Table S7: Experimental and coded values

Variables	−1	+1	L1	L2
Temp	rt	90	/	/
Addition	/	/	one-shot	portion-wise

S9 Central Composite Design - Optimization Phase

Table S8: CCD factors combination.

Entry	NaOH (equiv.)	CINN-Cl (equiv.)
1	2.0	0.586
2	2.0	2.0
3	2.0	2.0
4	1.0	1.0
5	2.0	2.0
6	2.0	3.414
7	2.0	2.0
8	3.414	2.0
9	0.586	2.0
10	3.0	1.0
11	1.0	3.0
12	3.0	3.0
13	2.0	2.0

Table S9: Experimental and coded values.

Variables	$-\alpha$	-1	0	$+1$	$+\alpha$
NaOH (equiv.)<br>CINN-Cl (equiv.)	0.586	1.0	2.0	3.0	3.414

S9.1 Estimated Coefficients Effects "Solid a" DS

Table S10: Estimated Coeff. for "solid a" DS as the ouput.

Factors	Effect	*p*-Value*
intercept	1.3441	/
**[NaOH]**	**0.3431**	**0.0023**
**[CINN]**	**0.3003**	**0.0047**
**[NaOH*CINN]**	**0.2858**	**0.0289**
**$[NaOH]^2$**	**-0.3933**	**0.0016**
**$[CINN]^2$**	**-0.1998**	**0.0395**

**Statistically significant at 95% of confidence level.*
*Factors in **bold** are significant ($p < 0.05$).*

S9.2 Estimated Coefficients Effects "Solid b" DS

Table S11: Estimated Coeff. for "solid b" DS as the ouput.

Factors	Effect	*p*-Value*
intercept	1.7172	/
[NaOH]	0.0047	0.9663
**[CINN]**	**0.6225**	**0.0006**
**[NaOH*CINN]**	**0.4664**	**0.0176**
$[NaOH]^2$	−0.2680	0.0519
**$[CINN]^2$**	**−0.3491**	**0.0187**

**Statistically significant at 95% of confidence level.*

*Factors in **bold** are significant ($p < 0.05$).*

S9.3 Estimated Coefficients Effects "Solid a" Weight

Table S12: Estimated Coeff. for "solid a" weight as the ouput.

Factors	Effect	*p*-Value*
intercept	170.76	/
[NaOH]	7.8460	0.7519
**[CINN]**	**−191.32**	**<.0001**
**[NaOH*CINN]**	**−134.53**	**0.0053**
**$[NaOH]^2$**	**155.68**	**0.0005**
**$[CINN]^2$**	**169.78**	**0.0003**

**Statistically significant at 95% of confidence level.*

*Factors in **bold** are significant ($p < 0.05$).*

S9.4 Estimated Coefficients Effects "Solid b" Weight

Table S13: Estimated Coeff. for "solid b" weight as the ouput.

Factors	Effect	*p*-Value*
intercept	537.34	/
**[NaOH]**	**89.038**	**0.0028**
**[CINN]**	**175.55**	**<.0001**
**[NaOH*CINN]**	**118.15**	**0.0039**
**$[NaOH]^2$**	**−153.15**	**0.0002**
**$[CINN]^2$**	**−141.68**	**0.0003**

**Statistically significant at 95% of confidence level.*

*Factors in **bold** are significant ($p < 0.05$).*

S10 Model validation

Model validation was performed by executing a control reaction at a selected "sweet spot", utilizing the quantities determined by the optimization software (Figure S21). The reagent loadings were precisely 2.64 equivalents of NaOH and 3.0 equivalents of CINN-Cl. The experimental protocol adhered strictly to the methodology employed during the optimization DoE. The subsequent table presents all relevant data for this validation test.

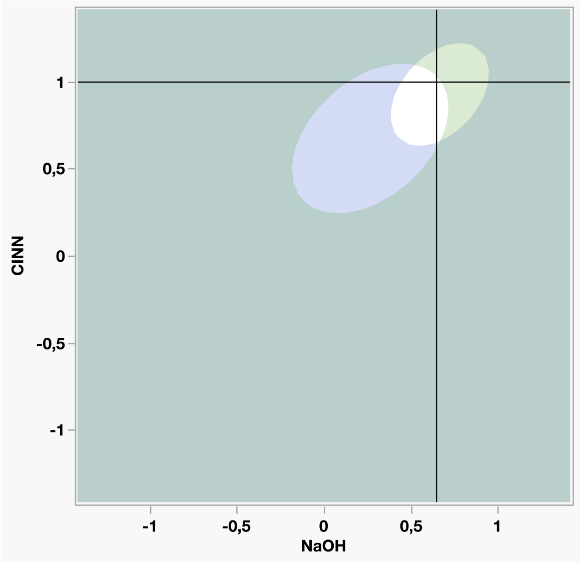

Figure S21: Contour plot showing the "sweet spot" in the proximity of the identified zone illustrated in Figure S4.

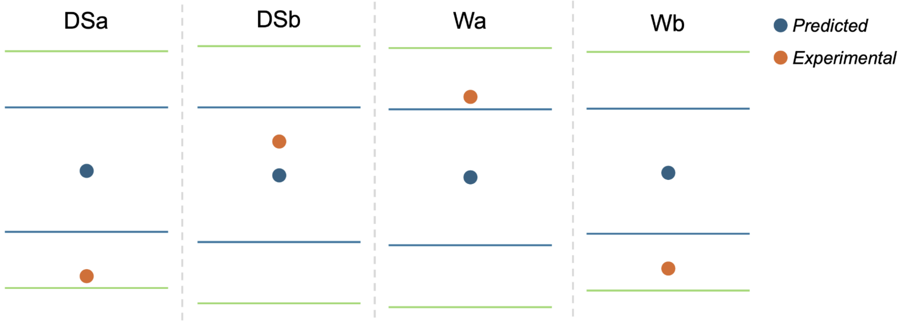

Figure S22: Predicted vs Experimental values compared with PIs (light green) and CIs (light blue); visual representation of Table S14 data.

Table S14: Estimated Coeff. for "solid b" weight as the ouput.

Response	Experiment.	Predicted	Upper PI	Lower PI	Upper CI	Lower CI
DSa	1.1648	1.6818	*2.2663*	*1.1067*	*1.9912*	*1.3818*
DSb	2.4012	2.1785	*3.0225*	*1.3433*	*2.6241*	*1.7417*
Wa	248.8	132.61	*319.76*	-55.42	*230.75*	*33.59*
Wb	514.0	638.96	*797.04*	*485.65*	*723.16*	*559.53*

PI: predictive interval; CI: confidence interval.

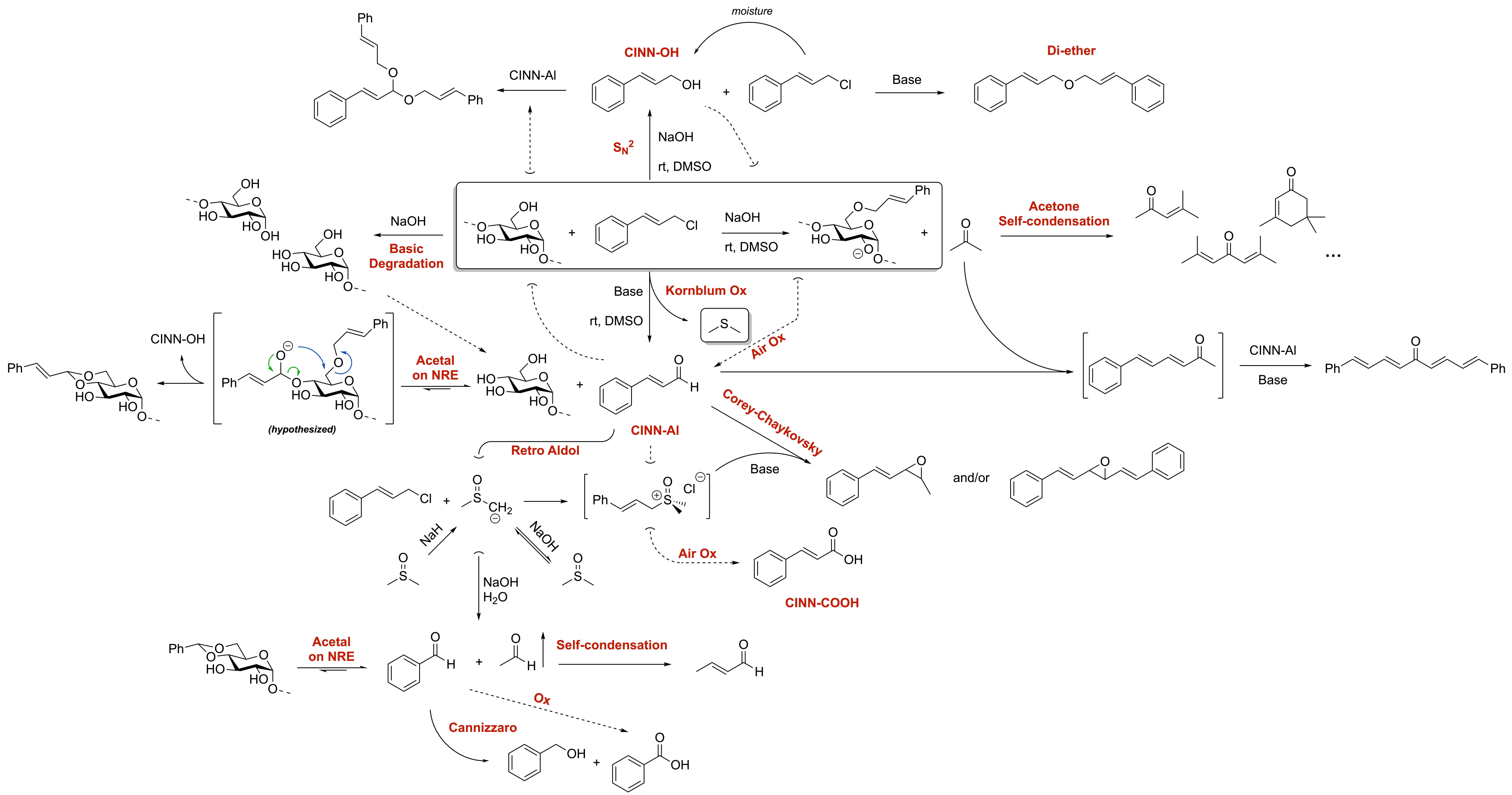

Figure S23: Proposed general scheme of both main side reactions and processes, that might happen within the studied system. In the rounded rectangle is reported the plain starch functionalization.

References

[1] L. Zoia, A. W. T. King, D. S. Argyropoulos, Molecular weight distributions and linkages in lignocellulosic materials derivatized from ionic liquid media, Journal of Agricultural and Food Chemistry 59 (2011) 829–838. doi:10.1021/jf103615e.

[2] M. Lenti, D. Parisi, P. Raffa, Starch benzoylation in supercritical co2. a novel sustainable route towards biodegradable hydrophobic polymeric materials, Carbohydrate Polymer Technologies and Applications 7 (2024) 100483. doi:10.1016/j.carpta.2024.100483.

[3] A. Rasouli, Y. Jamali, E. Tajkhorshid, O. Bavi, H. N. Pishkenari, Mechanical properties of ester- and ether-dphpc bilayers: A molecular dynamics study, Journal of the Mechanical Behavior of Biomedical Materials 117 (2021) 104386. doi:10.1016/j.jmbbm.2021.104386.

[4] C. Chi, Y. He, W. Jiao, H. Wang, X. Tan, Hierarchical structural transformation of corn starch in naoh solution at room temperature, Industrial Crops and Products 178 (2022) 114672. doi:10.1016/j.indcrop.2022.114672.
